# A systematic review and meta-analysis of gestational diabetes mellitus and mental health among BAME populations

**DOI:** 10.1016/j.eclinm.2021.101016

**Published:** 2021-07-14

**Authors:** Gayathri Delanerolle, Peter Phiri, Yutian Zeng, Kathleen Marston, Nicola Tempest, Paula Busuulwa, Ashish Shetty, William Goodison, Hemananda Muniraman, Georgia Duffy, Kathryn Elliot, Alison Maclean, Kingshuk Majumder, Martin Hirsch, Shanaya Rathod, Vanessa Raymont, Jian Qing Shi, Dharani K. Hapangama

**Affiliations:** a Oxford Brain Health Clinical Trials Unit, University of Oxford, United Kingdom; b University of Liverpool, United Kingdom; c University College London Hospitals NHS Foundation Trust, United Kingdom; d University College London, United Kingdom; e Southern Health NHS Foundation Trust, United Kingdom; f Liverpool Women's Hospital NHS Foundation Trust, United Kingdom; g School of Primary Care, Population Sciences and Medical Education, Faculty of Medicine, University of Southampton, United Kingdom; h University of Manchester NHS Foundation Trust, United Kingdom; i Southern University of Science and Technology, United Kingdom; j Alan Turing Institute, United Kingdom; k Department of Psychiatry, University of Oxford, United Kingdom; l Department of Pediatrics, Creighton University Medical School, United Kingdom

**Keywords:** Gestational diabetes mellitus, BAME, Mental Health, Women's Health and Wellbeing

## Abstract

**Background:**

Gestational diabetes mellitus (GDM) is a common complication of pregnancy and is associated with an increased risk of mental health (MH) disorders including antenatal and postnatal depression (PND), anxiety and post-traumatic-stress-disorder (PTSD). We hypothesized GDM and MH disorders will disproportionately affect individuals from Black, Asian and Minority Ethnic backgrounds.

**Methods:**

A systematic methodology was developed, and a protocol was published in PROSPERO (CRD42020210863) and a systematic review of publications between 1st January 1990 and 30th January 2021 was conducted. Multiple electronic databases were explored using keywords and MeSH terms. The finalised dataset was analysed using statistical methods such as random-effect models, subgroup analysis and sensitivity analysis. These were used to determine odds ratio (OR) and 95% confidence intervals (CI) to establish prevalence using variables of PND, anxiety, PTSD and stress to name a few.

**Findings:**

Sixty studies were finalised from the 20,040 data pool. Forty-six studies were included systematically with 14 used to meta-analyze GDM and MH outcomes. A second meta-analysis was conducted using 7 studies to determine GDM risk among Black, Asian and Minority Ethnic women with pre-existing MH disorders. The results indicate an increased risk with pooled adjusted OR for both reflected at 1.23, 95% CI of 1.00–1.50 and 1.29, 95% CI of 1.11–1.50 respectively.

**Interpretation:**

The available studies suggest a MH sequalae with GDM as well as a sequalae of GDM with MH among Black, Asian and Minority Ethnic populations. Our findings warrant further future exploration to better manage these patients.

**Funding:**

Not applicable

**Research in context**

**Evidence before this study**

Research of the gestational diabetes mellitus (GDM) and mental health (MH) sequalae is limited, especially among Black, Asian and Minority Ethnic (BAME) women. Evidence before this study is primarily cross-sectional in nature with small sample sizes where the primary focus is on non-BAME populations. Therefore, the generalisability of the findings to BAME patients remain limited. Similarly, cultural differences and barriers to access clinical care for BAME women with GDM and mental illness in general appears to be problematic and remain unresolved.

**Added value of this study**

This systematic review and meta-analysis demonstrate a number of MH symptomatologies and/or psychiatric comorbidities associated with GDM patients from BAME. To our knowledge this is the first study identifying and reporting the bidirectional relationship between GDM and MH among BAME patients.

**Implications of all the available evidence**

This systematic review demonstrates a complex bidirectional relationship between MH and GDM where further research is needed to establish the precise pathophysiology. Cultural adaptations could be a useful approach to consider when developing future diagnosis and treatment interventions to support the MH and GDM care needs for BAME patients. Additionally, a key step to improve patient reported outcomes would be to promote literacy of the disease *sequalae* among all stakeholders.

Alt-text: Unlabelled box

## 1 Introduction

Gestational Diabetes Mellitus (GDM) is a common medical disorder among pregnant women, affecting approximately 14% of pregnant women [1]. GDM is defined as a transitory form of glucose intolerance, induced by insulin resistance and pancreatic β-cell dysfunction during pregnancy [2]. It is a complex maternal health condition associated with short and long‐term complications. Risk factors associated with GDM include family history of diabetes, smoking, ethnicity [1] advancing maternal age and polycystic ovarian syndrome [3] (Fig. 2). In particular, obesity could induce chronic background insulin resistance, mediating metabolically induced inflammation [4] along with placental hormones that contribute to a state of insulin resistance. Thus, obese women are particularly susceptible to GDM [1]. GDM is commonly associated with an increased risk of type 2 Diabetes Mellitus (DM) in later life with risk factors for both conditions broadly similar [3]. As observed in type 2 diabetes [3], a major determinant for developing GDM is ethnic origin. 15% of women with a South Asian heritage may develop this complication whilst Caucasian women may only be affected in 3% of cases [3]. The presence of multiple risk factors does not reliably predict the risk of incidence of GDM [3]. GDM has multiple adverse implications for both mother and infant including hypertension, polyhydramnios and preterm labor in mother, and fetal macrosomia, birth injury, respiratory distress and hypoglycemia in the infant. Long-term consequences including development of type 2 DM and cardiovascular disease in women with GDM and metabolic syndrome in infants of mothers with GDM have also been reported [5,6].

A growing body of literature suggests the association between GDM and the subsequent development of mental health (MH) symptomatologies, notably depression and anxiety [5,7] with pooled prevalence of depression particularly being reported at 28%, although pathophysiological aspects remain unclear. Women with GDM are 2 to 4 times more likely to develop depression in the antenatal or postnatal periods in comparison to those without GDM [8,9,10]. The World Health Organisation's (WHO) ‘Women's Health Report’ published in 2016 demonstrates a higher incidence of MH issues amongst women in the reproductive ages (18 to 49 years) although, the data for MH *sequalae* associated with GDM is lacking [11].

Diagnosis and treatments for GDM women from Black, Asian and Minority Ethnic (BAME) backgrounds that report MH symptomatologies or have psychiatric conditions, remain non-specific. Pregnancy associated hormonal changes may attribute to emotional distress based on patient reported outcomes [12]. There are various forms of psychological distress such as diabetes-specific emotional distress, defined as negative emotions or fear related to lived experiences and coping mechanisms [13,14]. Alterations to mood could be attributed to hypothalamic-pituitary-adrenal axis dysfunction [6,15]. There is some evidence to support an association between GDM and the onset of MH disorders, although this relationship could be bi-directional [5,7,15]. Despite unclear inflammatory pathways, elevated levels of pro-inflammatory cytokines have been observed in both GDM and depression patients [15]. Given that pregnancy is commonly associated with heightened emotions, an additional GDM diagnosis could increase psychological strain [16]. Psychosocial dynamics such as social media could further impact mental and physical health of these women.

Both, MH conditions and GDM have been demonstrated to disproportionately affect those from BAME communities [17]. BAME women may endeavor additional challenges with accessing culturally responsive antenatal care associated with GDM and MH support due to a multitude of reasons albeit, perceptions and stigmatization being primary factors. Prospective data associated with the potential sequalae shared between GDM and MH remains limited [18].

Challenges around undiagnosed psychiatric conditions such as post-traumatic stress disorders (PTSD) and schizophrenia could result in exacerbation of secondary conditions such as GDM and vice versa [19]. This may be heightened among the BAME population experiencing racial discrimination [19] or inequalities leading to mistrust of healthcare services. It is reported, stigmatization faced by certain ethnic minorities may result in the worsening of emotional wellbeing leading to barriers [19]. These issues may impact the therapeutic rapport between the patient and healthcare professionals [19]. It has been reported that BAME women are less likely to receive the culturally responsive MH support compared to Caucasian women [20]. This may pose severe consequences as untreated depression in pregnancy has been associated with adverse pregnancy outcomes [21]. Pregnancies complicated by GDM as well as MH symptomatologies would be deemed high-risk and require specialist support from multiple clinical specialists of endocrinologists, psychiatrists and obstetricians.

A systematic review and meta-analysis was conducted to explore the MH impact on GDM patients and vice versa to better understand the disease sequalae, which reports the currently available knowledge and evaluate any practice gaps.

## 2 Methods

A systematic methodology (Fig. 2) was developed to determine the bi-directional relationship between GDM and MH patients. A systematic protocol was designed, peer reviewed and published on PROSPERO; (CRD42020210863).

**Fig. 1:**
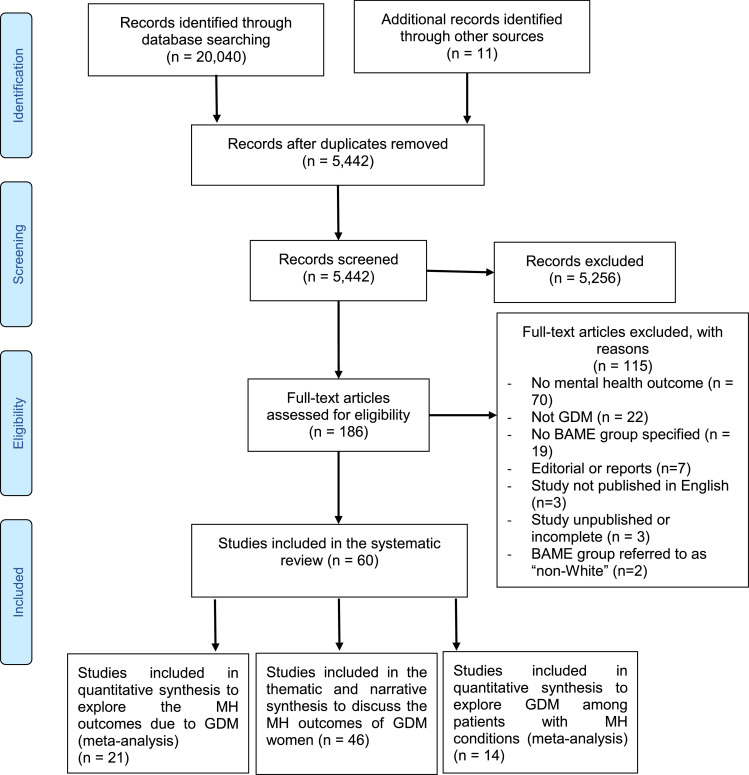
PRISMA flow diagram.

The search strategy comprised of the use of multiple MeSH terms and key words such as *Depression, Anxiety, Mental Health and Gestational Diabetes, Mental Health in Gestational Diabetes in BAME, Biopolar and Psychosis.* Further details are provided in S Fig. 1. The primary aim of this study was to assess the prevalence of the GDM and all MH symptomatologies reported and psychiatric comorbidities among BAME women.

The aims of this study would be to determine the prevalence of the bidirectional relationship between MH outcomes among GDM patients within the BAME population.

### 2.1 Study eligibility criteria

All randomised controlled trials (RCTs) and non-RCTs reporting MH symptoms and/or psychiatric comorbidities as described above were included. Studies published in English from the 1st of November 1995 to 30th November 2020 were included into this study.

### 2.2 Data extraction and synthesis

Multiple databases were used, including PubMed, PROSPERO, EMBASE, ProQuest and Science direct. Predefined clinical variables of depression, anxiety, stress, schizophrenia and PTSD were used within the search strategy. A full list of the predefined clinical variables are shown in Table 8. An evidence synthesis protocol has been provided for further information as Supplementary Fig. 1. The data extraction process was documented using PRISMA (Fig. 1). The data extraction and refinement processes were completed using Endnote and Microsoft Excel by 4 reviewers. An independent reviewer was used to evaluate the dataset prior to the statistical analysis.

Studies included within this study have been categorised as per the key characteristics and synthesised based on details such as relative risks (RRs), odds ratios (ORs), prevalence risks (PRs), media, mean differences (MDs) and their 95%CI were collated as part of the data synthesis method. Prevalence tables would be generated to demonstrate the subgroup categories such as geographical location, ethnicity and race. Systematically included studies that have insufficient statistical data reported that is needed for a meta-analysis would be narratively analysed from a patient, family, society, clinician and healthcare provider perspective. The narration would synthesis and report any potential barriers to the any author identified themes and sub-themes, where possible. A thematic content analysis would be undertaken to identify fundamental common categories which may include professional clinical guidelines and recommendations to report any commonality.

The outcome assessments for depression or anxiety were based on Edinburgh Postnatal Depression Scale(EPDS), Perceived Stress Scale (PSS), centre for Epidemiologic Studies Depression Scale (CES-D) and Patient Health Questionnaire (PHQ-9) [22] of the ACHOIS group indicated the RR and 95% CI whilst others studies reported ORs and 95%CI. For the studies that reported RR, the formula below was used to determine the unadjusted and adjusted analysis. The use of the term of adjusted analysis here is in the context of the use of adjusted and crude OR [23].

$$\hat{RR}=\frac{OR}{1-P_{0}+P_{0}*OR}$$

Where $P_{0}$ is the general prevalence of GDM among pregnancies. The extracted quantitative data was synthesised as per the methodology protocol (S Fig 1) and analysed using the meta-analysis. Data gathered systematically were synthesised narratively. Key clinical variables used within the study are reported within Table 2. Heterogeneity was assessed by I^2^ and other investigated tools and further by using a subgroup analysis whilst the Egger's test was used to evaluate publication bias.

### 2.3 Outcomes

Primary outcome of interest is to report the prevalence of the bidirectional relationship between GDM and MH among BAME patients. Additionally, the following outcomes would be reported;

1. PTSD among BAME patients with GDM
2. PND among BAME patients with GDM
3. Anxiety among BAME patients with GDM
4. Stress among BAME patients with GDM
5. GDM among BAME patients with a pre-existing depression diagnosis
6. Psychological distress among BAME patients
7. MH assessment used within the BAME GDM population
8. Psychiatric comorbidities used within the BAME GDM population

### 2.4 Heterogeneity assessment

The methodological heterogeneity was assessed with forest plots and chi-quare tests (*P*< 0.05 demonstrates a significant heterogeneity) as well as I^2^. I^2^ is representative of the percentage of variability observed across the pooled studies within the meta-analysis that could attribute to the apparent heterogeneity. In the presence of I^2^ of >50% statistic demonstrating moderate to substantial heterogeneity.

### 2.5 Risk of bias (Quality assessment)

Most studies included in this review were cross-sectionally designed. All literature identified and reported have been appraised individually against the predefined variables critically. Independent reviewers indicated methodological quality and rigor. The Newcastle-Ottawa-Scale (NOS) was used to determine the quality of the studies included within the meta-analysis [Table 1a, 1b, 1c and 1d]. This was furthered by the application of the refinement protocol (S Fig. 1) where all studies included in both meta-analyses were evaluated against the eligibility criteria which demonstrated the scientific basis of the analysis conducted.

**Table 1a:** Characteristics of the studies included in the systematic review.

Study ID	Author	Study type	Sample size	Exposure	Outcome	Outcome assessment	NOS Score
1	Abdollahi, F et al	Longitudinal Cohort Study	1449	Gestational Diabetes	Post-partum depression	EPDS≥12	****** (6)
2	Ahmed, Anwar E et al	Cross-sectional Study	438	Gestational Diabetes	Stress	PSS≥20	******* (7)
3	Bandyopadhyay, M. et al	Qualitative Study	17	Gestational Diabetes	Women's’ response to GDM diagnosis and experiences managing the condition.	Interview	****** (6)
4	Beka, Q. et al.	Cohort Study	326,273	Gestational Diabetes	2) Mental illness postpartum	Medical records.	****** (6)
5	Beka, Q. et al	Retrospective Cohort Study	253,911	medical records of mental health	Gestational Diabetes	Alberta Perinatal Health Program.	******* (7)
6	Borgen, I. et al	Cross-sectional Study	217	Gestational Diabetes	Depression	EPDS≥7	****** (6)
7	Bowers, K. et al	Cohort Study	121,260	Depression	Gestational Diabetes	Medical records.	******* (7)
8	Byrn, Mary et al	Cross-sectional Study	135	Gestational Diabetes	Depression	EPDS≥12	******* (7)
9	Carson, L. D. et al	Qualitative Study	97	Pregnant women and receiving care from a tribal healthcare clinic.	The perceptions and concerns regarding diabetes mellitus during pregnancy among American Indian women.	Questionnaire	****** (6)
10	Catherine, K. I. M. et al	Cross-sectional Study	7065	Gestational Diabetes	Poor health and mental distress.	NHIS six item Non-Specific Distress Battery	****** (6)
11	Chazotte C. et al	Case-control Study	30	Gestational Diabetes	Depression	CES-*D* ≥ 16	****** (6)
12(a)	Clark, C. E. et al	Case-control Study	1439	Depression prior to pregnancy	Gestational diabetes	OGTT	******* (7)
12(b)	Clark, C. E. et al	Case-control Study	1439	Gestational Diabetes	Depression	charts within six months of delivery	******* (7)
13(a)	Crowther, C. A. et al	Randomised Clinical Trial	1000	dietary advice, blood glucose monitoring, and insulin therapy	The effect of the intervention on depression	EPDS≥12	******* (7)
13(b)	Crowther, C. A. et al	Randomised Clinical Trial	1000	dietary advice, blood glucose monitoring, and insulin therapy	The effect of the intervention on anxiety	Spielberger State–Trait Anxiety Inventory≥15	******* (7)
14	Dahlen, H. G. et al	Cohort Study	3092	Depression.	Gestational Diabetes	Clinical data from first antenatal visit, through to discharge of mother and baby from the hospital.	****** (6)
15	Damé, P. et al	Cross-sectional Study	820	Gestational Diabetes	Depression	EPDS	****** (6)
16	Dayyani, I. et al	Qualitative Study	11	Gestational Diabetes	The experiences of ethnic minority women with Gestational Diabetes.	Interviews	***** (5)
17	Dickson, L. M. et al	Qualitative Study	10	All women were black African with GDM.	To identify the personal challenges, experiences and health decisions following a GD diagnosis.	The Diabetes Conversation Map educational instrument	****** (6)
18	Draffin, C. R. et al	Qualitative Study	19	GDM or a history of GDM	Identifying the concerns, needs and knowledge of women with GDM.	According to topic, allowing further identification of sub-themes.	****** (6)
19	Draffin, Claire R. et al	Randomised Controlled Trial	150	GDM	Anxiety	STAI	******* (7)
20	Feig, Denice S. et al	Case-control Study	4274	history of a gestational diabetes	Self-perceived status	Post-partum Health Questionnaire	***** (5)
21	Ge, L. et al	Qualitative Study	17	All women had Gestational Diabetes.	Beliefs about illness and health amongst women 22with Gestational Diabetes in South East Asia.	Interview	******* (7)
22	Ge, L. et al	Qualitative Study	62	All women had Gestational Diabetes	The experiences of living with Gestational Diabetes for women in China.	Interview	******* (7)
23	Ghaffari, F. et al	Qualitative Study	25	Gestational Diabetes	Factors affecting treatment compliance for women with gestational diabetes in Iran.	Interview	****** (6)
24	Guo, Jia et al	Mixed-Methods Study	323	Gestational Diabetes	Barriers to blood glucose level monitoring. This included depressive symptomatology.	CES-*D* ≥ 16	******* (7)
25(a)	Hinkle, S. N. et al	Longitudinal Study	2477	1) Depression in the 1st and 2nd trimesters	Gestational diabetes	medical record	******* (7)
25(b)	Hinkle, S. N. et al	Longitudinal Study	2477	2) Gestational Diabetes	Postpartum depression	EPDS≥10	******* (7)
26	Hirst JE et al	Qualitative Study	34	Gestational Diabetes	Attitudes and health behaviours in women with GDM.	Nvivo 9 (QSR International).	******* (7)
27	Hjelm K et al	Qualitative Study	14	Gestational Diabetes	The beliefs about health and illness	Interview	***** (5)
28	Hjelm, K et al	Qualitative Study	9	Gestational Diabetes	Beliefs about health, illness and healthcare in migrant women with GDM	Interview	***** (5)
29	Hjelm, K. et al	Qualitative Study	27	All women had GDM.	The beliefs about health and illness between women born in Sweden and the Middle East who developed gestational diabetes.	Interview	***** (5)
30	Huang T et al	Cross sectional Study	1686	GDM	Postpartum depression - measured at 6 months post-partum	EPDS>13	***** (5)
31(a)	Hui, A. L. et al	Mixed-Methods Study	30	Gestational Diabetes	Anxiety	PSS	******* (7)
31(b)	Hui, A. L. et al	Mixed-Methods Study	30	Gestational Diabetes	Anxiety	Pregnancy Anxiety Scale.	******* (7)
31(c)	Hui, A. L. et al	Mixed-Methods Study	30	Gestational Diabetes	Anxiety	STAI	******* (7)
32	Hui, Amy Leung et al	Qualitative Study	30	All women had GDM.	Understanding the barriers and coping strategies for women with GD to follow dietary advice	Interview	******* (7)
33	Jirojwong, S. et al	Qualitative Study	19	All South East Asian migrant women with GDM.	Migrant women's experiences of a GD diagnosis.	Interview	****** (6)
34	Katon, J. G. et al	Cross-sectional study	2398	GDM	Antenatal depression	PHQ-9	****** (6)
35	Kim C et al	Case-control Study	1445	Gestational Diabetes	Depression	CES-*D*>10	******* (7)
36	Kozhimannil, K.B et al	Retrospective Cohort Study	11,024	Gestational diabetes, not taking insulin	Depression	Depression or a prescription drugs.	******* (7)
37	Lapolla, A. et al	Qualitative Study	286	All participants with GDM.	Quality of Life in women with GD	Diabetes Attitudes, Wishes and Needs survey	****** (6)
38	Lara-Cinisomo, S. et al	Cohort Study	34	Gestational Diabetes (*n* = 5)	Postnatal Depression	EPDS>10	***** (5)
39	Larrabure-Torrealva, G. T. et al	Cross sectional Study	1300	Depression	Gestational diabetes	OGTT	******* (7)
40	Lau, Y. et al	Longitudinal Study	361	Gestational Diabetes	Postpartum Depression	EPDS >9	****** (6)
41	Liu, C. H. et al	Cohort Study	3738	Gestational Diabetes	Postpartum Depression	PRAMS	****** (6)
42	Mak, J. K. L. et al	Cohort Study	1449	Gestational Diabetes	Depression	EPDS	****** (6)
43	McCloskey, L. et al	Qualitative Study	59	GDM	Providers’ and patients’ experiences and challenges related to GDM.	Interview	******* (7)
44	Mensah, Gwendolyn Patience et al	Qualitative Study	15	Nurse midwives involved with the care of women with GD. Women attending the military hospital in Ghana with GD.	Experiences regarding the care, treatment and management of Gestational Diabetes in Ghana.	Interviews	****** (6)
45	Monk, C. et al	Cohort Study	4161	Gestational Diabetes	Stress	PSS	****** (6)
46	Muhwava, L. S. et al	Qualitative Study	35	a history of GD	Experiences of lifestyle change among women with GD	Interviews	****** (6)
47	Natasha, K. et al	Observational Study	748	Gestational Diabetes	Depression	MADRS scale.	***** (5)
48	Neufeld, H. T. et al	Qualitative Study	29	GD	The food perceptions and concerns of Gestational Diabetes among Aboriginal people.	Interview	***** (5)
49	Nicklas, J. M. et al	Observational Study	71	Gestational Diabetes	Postpartum depression	EPDS> 9	****** (6)
50	Nielsen, K. K. et al	Qualitative Study	19	Gestational Diabetes	The experiences of women with Gestational Diabetes.	Interview	***** (5)
51	Nikakhlagh, Mahnaz et al	Mixed-Methods Study	24	Gestational Diabetes	Quality of life.	EMSQ and WHOQoL (short form)	******* (7)
52	O'Reilly, S. L. et al	Randomised controlled trial	573	gestational diabetes	Depression	PHQ-9>20	******* (7)
53	Packer, C. H. et al	Retrospective Cohort Study	170,572	Gestational Diabetes	Depression	Depression was medically diagnosed	******** (8)
54	Parsons, J. et al	Qualitative Study	50	Gestational Diabetes	The experiences of Gestational Diabetes and Gestational Diabetes care.	Interview	***** (5)
55	Ragland, Denise et al	Observational Study	50	Gestational Diabetes	Depression	Beck Depression Inventory>13	****** (6)
56	Razee, H. et al	Qualitative Study	57	All women had a history of Gestational Diabetes in the previous 6–36 months.	The experiences, beliefs, support and environmental influences related to gestational diabetes.	Interview	****** (6)
57	Reid, J. et al	Qualitative Study	10	The woman had a history of Gestational Diabetes (*n* = 8) or had been exposed to diabetes in utero (*n* = 2)	The experiences of indigenous women with a gestational diabetes diagnosis.	Interview	***** (5)
59	Schmidt, C. B. et al	Cohort Study	100	All women had Gestational Diabetes	Depression	PHQ-9>12	****** (6)
60	Shokrpour, M. et al	Case-control study	170	This was a case-control study. 85 women had GDM, 85 did not.	Postpartum Depression	EPDS	******* (7)
61	Siad, Fartoon M. et al	Qualitative Study	10	All women had Gestational Diabetes.	The experiences of Gestational Diabetes amongst women from East Africa.	Interview	***** (5)
62	Silveira, M. L. et al	Prospective cohort study	1308	Perceived stress	Gestational Diabetes	plasma glucose, OGTT	****** (6)
63	Walmer, R. et al	Case-control Study	18,109	GDM	mental health disorders	electronic medical records	******* (7)
64	Wilson, B. L. et al	Correlation study	3655	Depression, stress and physical abuse	Gestational Diabetes	PRAMS self-report	****** (6)
65(a)	Wilson, C. A. et al	Cohort Study	12,239	GDM	1) Antenatal mental health disorders	Medical records.	******* (7)
65(b)	Wilson, C. A. et al	Cohort Study	12,239	Preconception mental health disorders	2) Gestational Diabetes Diagnosis	glucose	******* (7)
66	Yang, X. et al	Randomised Controlled Trial	700	Gestational Diabetes	Depression	PHQ	****** (6)
67	Youngwanichsetha S et al	Qualitative Study	30	Gestational Diabetes.	Experiences of blood glucose monitoring for Thai women with Gestational Diabetes.	Interviews	****** (6)
68	Zadeh, N. N. et al	Case-control study	100	Gestational Diabetes	General health - depression and anxiety measures in this questionnaire.	GHQ	****** (6)
69	Zulfiqar, Tehzeeb et al	Qualitative Study	23	Women with a history of Gestational Diabetes.	The barriers and facilitators to a healthy lifestyle following a diagnosis with Gestational Diabetes.	Interview	***** (5)

*Quality of the included cross-sectional studies was measured using the modified Newcastle-Ottawa Measurement Scale specific for Cross-sectional studies.

We rated the quality of the studies (good, fair and poor) by allocating each domain with stars in this manner:.

• A Good quality score was awarded 3 or 4 stars in selection, 1 or 2 in comparability, and 2 or 3 stars in outcomes.

• A Fair quality score was awarded 2 stars in selection, 1 or 2 stars in comparability, and 2 or 3 stars in outcomes.

• A Poor quality score was allocated 0 or 1 star(s) in selection, 0 stars in comparability, and 0 or 1 star(s) in outcomes domain in line with the NOS guidelines.

**Table 1b:** Quality assessment of the included studies using the Newcastle Ottawa Scale.

	Selection (S)				Comparability ©		Exposure/Outcome E/O			Sub Total assessment			
	1	2	3	4	1a	1b	1	2	3	*S*^+^	C^&^	E/O^&^	Conclusion
Abdollahi, F et al	*	*	No	*	*	*	No	*	*	Good	Good	Good	Good
Ahmed, Anwar E et al	*	*	*	*	*	*	*	*	*	Good	Good	Good	Good
Bandyopadhyay, M et al	*	*	*	*	*	*	*	*	*	Good	Good	Good	Good
Beka, Q. et al	*	*	*	*	*	*	*	*	*	Good	Good	Good	Good
Beka,Q et al	*	*	*	*	*	*	*	*	*	Good	Good	Good	Good
Borgen, I. et al	*	*	No	*	*	*	*	*	*	Good	Good	Good	Good
Bowers, K et al	*	*	*	*	*	*	*	*	*	Good	Good	Good	Good
Byrn, Mary et al	*	*	*	*	*	*	*	*	*	Good	Good	Good	Good
Carson, L et al	*	*	No	*	*	*	*	No	*	Good	Good	Good	Good
Catherine, K.I.M. et al	*	*	*	*	*	*	*	*	*	Good	Good	Good	Good
Chazotte C., et al	*	*	*	*	*	*	*	*	*	Good	Good	Good	Good
Clark, C.E. et al	*	*	*	*	*	*	*	*	*	Good	Good	Good	Good
Crowther, C. A. et al	*	*	*	*	*	*	*	*	*	Good	Good	Good	Good
Dahlen, H.G. et al	*	*	*	*	*	*	*	*	*	Good	Good	Good	Good
Damé, P. et al	*	No	No	*	No	*	*	*	*	Fair	Good	Good	Fair
Dayyani, I. et al	No	No	*	*	*	*	*	No	*	Fair	Good	Good	Fair
Dickson, L.M et al	No	*	*	*	*	*	*	No	*	Good	Good	Good	Good
Draffin, C.R. et al	No	No	*	*	No	*	*	*	*	Fair	Good	Good	Fair
Draffin, C.R. et al	No	No	*	*	No	*	*	*	*	Fair	Good	Good	Fair
Feig, Denice S. et al	*	*	No	No	*	*	*	No	*	Fair	Good	Good	Good
Ge, L. et al	*	No	*	*	*	*	*	*	*	Good	Good	Good	Good
Ge, L. et al	*	No	*	*	*	*	*	*	*	Good	Good	Good	Good
Ghaffari, F. et al	*	*	*	*	*	*	*	*	*	Good	Good	Good	Good
Guo, Jia. Et al	*	*	*	*	*	*	*	*	*	Good	Good	Good	Good
Hirst JE, et al	*	*	*	*	*	*	*	*	*	Good	Good	Good	Good
Hinkle, S., et al	*	No	*	No	*	*	*	*	*	Fair	Good	Good	Fair
Hjelm, K. et al	*	No	*	No	*	*	*	No	No	Fair	Good	Poor	Poor
Hjelm, K. et al	No	No	*	*	*	*	*	*	*	Fair	Good	Good	Good
Hjelm, K. et al	No	No	*	*	*	*	*	*	*	Fair	Good	Good	Good
Huang T, et al	*	*	*	*	*	*	*	*	*	Good	Good	Good	Good
Hui, A. L. et al	*	*	*	*	*	*	*	*	*	Good	Good	Good	Good
Hui, A.L et al	*	*	*	*	*	*	*	*	*	Good	Good	Good	Good
Jirojwong, S. et al	No	*	*	*	*	*	*	No	*	Good	Good	Good	Good
Katon, J. G. et al.	*	*	*	*	*	*	*	*	*	Good	Good	Good	Good
Kim, C. et al	*	*	*	*	*	*	*	*	*	Good	Good	Good	Good
Kozhimannil, K.B et al	*	*	*	*	*	*	*	*	*	Good	Good	Good	Good
Lapolla, A. et al	*	*	*	*	*	*	*	*	*	Good	Good	Good	Good
Lara-Cinisomo, S. et al	No	*	*	*	*	*	*	*	*	Good	Good	Good	Good
Larrabure-Torrealva, G. T.	*	*	*	*	*	*	*	*	*	Good	Good	Good	Good
Lau, Y. et al	*	*	*	*	*	*	*	*	*	Good	Good	Good	Good
Liu, C. H. et al	*	*	*	*	*	*	*	*	*	Good	Good	Good	Good
Mak, J. K. L. et al	*	*	*	*	*	*	*	*	*	Good	Good	Good	Good
McCloskey, L. et al	No	*	*	*	*	*	*	*	*	Good	Good	Good	Good
Mensah, Gwendolyn Patience et al	No	*	*	*	*	*	*	*	*	Good	Good	Good	Good
Monk, C. et al	*	*	*	*	*	*	*	*	*	Good	Good	Good	Good
Muhwava, L. S. et al	*	*	*	*	*	*	*	*	*	Good	Good	Good	Good
Natasha, K. et al	*	*	*	*	*	*	*	*	*	Good	Good	Good	Good
Neufeld, H.T. et al	No	*	No	*	*	*	*	*	*	Fair	Good	Good	Fair
Nicklas, J. M. et al	*	*	No	*	*	*	*	*	*	Good	Good	Good	Good
Nielsen, K.K. et al	No	No	*	*	*	*	*	*	*	Fair	Good	Good	Fair
Nikakhlagh, Mahnaz et al	No	*	*	*	*	*	*	*	*	Good	Good	Good	Good
O'Reilly, S. L. et al	*	*	*	*	*	*	*	*	*	Good	Good	Good	Good
Packer, C. H. et al	*	*	*	*	*	*	*	*	*	Good	Good	Good	Good
Parsons, J.et al	No	*	*	*	*	*	*	*	*	Good	Good	Good	Good
Ragland, Denise et al	*	*	*	*	*	*	*	*	*	Good	Good	Good	Good
Razee, H. et al	*	*	No	*	*	*	*	No	*	Good	Good	Good	Good
Reid, J. et al	No	*	No	*	*	*	*	*	*	Fair	Good	Good	Fair
Schmidt, C. B. et al	No	*	No	*	*	*	No	*	*	Fair	Good	Good	Good
Shokrpour, M. et al	*	*	*	*	*	*	*	*	*	Good	Good	Good	Good
Siad Fartoon, M. et al	No	*	No	*	*	*	*	No	*	Fair	Good	Good	Fair
Walmer, R. et al	*	*	*	*	*	*	*	*	*	Good	Good	Good	Good
Wilson, B.L. et al	*	*	*	*	*	*	*	*	*	Good	Good	Good	Good
Wilson, C. A.	*	*	*	*	*	*	*	*	*	Good	Good	Good	Good
Yang, X. et al	*	*	*	*	*	*	*	*	*	Good	Good	Good	Good
Youngwanichsetha S, et al	No	*	*	*	*	*	*	*	*	Good	Good	Good	Good
Zadeh, N. N. et al	*	*	*	*	*	*	No	*	*	Good	Good	Good	Good
Zulfiqar, Tehzeeb et al	No	*	No	*	*	*	*	*	*	Fair	Good	Good	Fair

**Table 1c:** Summary of the meta-analyses.

Exposure	Outcome	k	Odds Ratio	95%CI	z-value	*p*-value	Heterogeneity I^2^(%)
**Women with GDM compared to women without GDM**							
GDM	Depression	**12**	**1.22**	**0.94 to 1.57**	**1.50**	**0.13**	**72.97**
GDM	Anxiety	**2**	**1.09**	**0.98 to 1.22**	**1.59**	**0.11**	**88.45**
GDM	Stress	**2**	**2.29**	**0.98 to 5.37**	**1.91**	**0.06**	**0**
**Women with depression compared to women without depression**							
Depression	GDM	**6**	**1.3**	**1.07 to 1.57**	**2.66**	**0.01**	**70.82**

**Table 1d:** Summary of the subgroup analysis.

						Between groups	
	k	Odds Ratio	95%CI	Q-value	*p*-value	Q-value	*p*-value
**Women with GDM suffered from Depression compared to women without GDM**							
**Type of OR**							
Adjusted OR	**12**	**1.22**	**0.94 to 1.57**	**40.69**	**0.00**		
Unadjusted OR	**8**	**1.38**	**1.09 to 1.73**	**20.47**	**0.00**		
						**0.49**	**0.48**
**Type of study**							
RCT study	**1**	**0.43**	**0.27 to 0.7**	**—**	**—**		
Epidemiology study	**11**	**1.32**	**1.06 to 1.65**	**59.68**	**0.01**		
						**16.94**	**0.00**
**Type of study (detailed)**							
RCT study	**1**	**0.43**	**0.27 to 0.70**	**—**	**—**		
Case-control study	**2**	**1.28**	**0.99 to 1.67**	**0.02**	**0.90**		
Cohort study	**4**	**0.90**	**0.73 to 1.10**	**0.12**	**0.73**		
Cross-sectional study	**2**	**1.13**	**0.88 to 1.46**	**2.49**	**0.48**		
Longitudinal cohort study	**1**	**2.16**	**1.51 to 3.08**	**1.01**	**0.31**		
Retrospective cohort study	**1**	**1.72**	**1.11 to 2.66**	**—**	**—**		
						**36.84**	**0.00**
**Ethnicity subgroup**							
Asian	**4**	**0.96**	**0.55 to 1.65**	**22.11**	**0.00**		
Black	**2**	**0.51**	**0.10 to 2.60**	**1.67**	**0.2**		
Hispanic	**2**	**1.40**	**1.15 to 1.70**	**0.00**	**1.00**		
						**3.00**	**0.22**

### 2.6 Terms of reference

We acknowledge and agree there is a difference between biological sex and/or gender. These terms have different meanings to various communities which also bares legal conformities. Equally, the use of these terms clinically could vary depending on the condition being explored. We respectfully, use the term ‘women’ in line with those who are pregnant with an unborn child as GDM is a pregnancy related complication that may or may not elicit a MH outcome such as depression and/or anxiety.

We acknowledge and agree that the term “BAME” may not be favoured by some. We would like to acknowledge all authors within this publication are from Black, Asian, and Minority Ethnic (BAME) as well as, Caucasian backgrounds. We acknowledge our own differing cultural backgrounds, religions, and beliefs. We respect and acknowledge all differing views and thoughts without any prejudice as race and ethnicity are complex aspects to discuss. This publication is not attempting to discuss the complexities around ethnicity and race but infer to the role it could play in the exploration of the bidirectional Gestational Diabetes Mellitus and Mental Health relationship. We have used the term “BAME” to be factually correct to report the evidence identified as, this is currently the legally accepted term in the UK although we acknowledge this may amend in the future both in the UK and globally.

### 2.7 Role of funding sources

Not applicable

## 3 Results

An initial search identified 20,040 studies, of which, those with limited discussions in relation to MH outcomes and of poor quality, were excluded, resulting in a final dataset of 69 studies (Table 1a) of which 46 studies were systematically included and thematically analysed (Table 2). These 46 studies included 26 qualitative [12,[22], [23], [24], [25], [26], [27], [28], [29], [30], [31], [32], [33], [34], [35], [36], [37], [38], [39], [40], [41], [42], [43], [44], [45], [46]], 17 cohort [7,18,21,[47], [48], [49], [50], [51], [52], [53], [54], [55], [56], [57], [58], [59], [60]], 6 controlled [61], [62], [63], [64], [65], [66], 2 randomised controlled trials [19,67] and 2 mixed methods [68,69] studies. Based on the eligibility criteria, and the quality assessment, 21 studies were selected for the meta-analysis.

**Table 2:** demonstrates the characteristics of the studies included within the Thematic and Narrative synthesis.

Study ID	Author	Study type	Sample size	Exposure	Outcome	Outcome assessment
1	Abdollahi, F et al	Longitudinal Cohort Study	1449	Gestational Diabetes	Post-partum depression	EPDS≥12
2	Ahmed, Anwar E et al	Cross-sectional Study	438	Gestational Diabetes	Stress	PSS≥20
3	Beka, Q. et al	Cohort Study	326,723	Gestational Diabetes	Mental illness	At least 1 hospitalization, outpatient visit or physician claim for an affective or anxiety disorder.
4	Borgen, I. et al	Cross-sectional Study	217	Gestational Diabetes	Depression	EPDS≥7
5	Byrn, Mary et al	Cross-sectional Study	135	Medical history of Gestational Diabetes	Depression	EPDS≥12
7	Chazotte C., et al	Case-control Study	30	Gestational Diabetes	Depression	CES-*D* ≥ 16
8	Crowther, C. A. et al	Randomized Clinical Trial	1000		Depression	EPDS≥12
9	Damé, P. et al	Cross-sectional Study	820	Gestational Diabetes	Depression	EPDS≥12
10	Feig, Denice S. et al	Case-control Study	4274	History of a gestational diabetes	Self-perceived status	Post-partum Health Questionnaire
11	Ge, L. et al	Qualitative Study	17	Gestational Diabetes	Illness and health	The interviews
12	Hirst JE, et al	Qualitative Study	34	Gestational Diabetes	Attitudes and health behaviours	Nvivo 9 (QSR International)
13	Hinkle, S., et al	Longitudinal Study	2477	Depression	Gestational Diabetes	OGTT
14	Hjelm, K. et al	Qualitative Study	9	Gestational Diabetes	Beliefs about health, illness and healthcare	The interviews
15	Hjelm, K. et al	Qualitative Study	27	Gestational Diabetes	The beliefs about health and illness	The interviews
16	Huang T, et al	Cross-sectional Study	1686	Gestational Diabetes	Perinatal Depression	EPDS≥12
17	Hui, A. L. et al	Mixed-Methods Study	30	Gestational Diabetes	Anxiety	PSS
19	Kozhimannil, K.B et al	Retrospective Cohort Study	11,024	Gestational Diabetes	Depression	Diagnosis of depression or a prescription drugs.
20	Lapolla, A. et al	Qualitative Study	286	Gestational Diabetes	Quality of Life	Diabetes Attitudes, Wishes and Needs survey assessed the quality of life
21	Lara-Cinisomo, S. et al	Cohort Study	34	Gestational Diabetes	Postnatal Depression	EPDS>10
22	Lau, Y. et al	Longitudinal Study	361	Gestational Diabetes	Postpartum Depression	EPDS>9
23	Liu, C. H. et al	Cohort Study	3738	Gestational Diabetes	Postpartum Depression	Pregnancy Risk Assessment Monitoring System survey
24	Mak, J. K. L. et al	Cohort Study	1449	Gestational Diabetes	Depression	EPDS
25	McCloskey, L. et al	Qualitative Study	59	Gestational Diabetes	Experiences and challenges related to GDM	The interviews
26	Monk, C. et al	Cohort Study	4161	Gestational Diabetes	Stress	PSS
27	Muhwava, L. S. et al	Qualitative Study	35	History of a gestational diabetes	lifestyle change among women with GD	The interviews
28	Natasha, K. et al	Observational Study	748	Gestational Diabetes	Depression	MADRS scale Mild depression (13–19), Moderate depression (20–34) and severe depression (35–60)
29	Nicklas, J. M. et al	Observational Study	71	Gestational Diabetes	Postpartum depression	EPDS>9
30	Nikakhlagh, Mahnaz et al	Mixed-Methods Study	24	Gestational Diabetes	Quality of life.	Enrich Marital Satisfaction Questionnaire (short form) and World Health Organization Quality of Life questionnaire (short form)
31	O'Reilly, S. L. et al	Randomized Controlled Trial	573	Gestational Diabetes	Depression	PHQ-9(Moderate depression was recorded as a score greater than ten and severe depression was noted as a score greater than 20.)
32	Packer, C. H. et al	Retrospective Cohort Study	170,572	Gestational Diabetes	Depression	Medically diagnosed
33	Ragland, Denise et al	Observational Study	50	Gestational Diabetes	Depression	Beck Depression Inventory>13
34	Razee, H. et al	Qualitative Study	57	Gestational Diabetes	The experiences, beliefs, support and environmental influences related to gestational diabetes.	The interviews
35	Sakeena, K et al	Cross-sectional Study	200	History of a gestational diabetes	Post-partum depression	The interviews
36	Schmidt, C. B. et al	Cohort Study	100	Gestational Diabetes	Depression	PHQ-9>12
37	Shokrpour, M. et al	Case-control study	170	Gestational Diabetes	Postpartum Depression	EPDS
39	Yang, X. et al	Randomized Controlled Trial	700	Gestational Diabetes	Depression	PHQ-9(10–14 was considered as minor depression and a score of 15 or higher was considered major depression)
40	Youngwanichsetha S, et al	Qualitative Study	30	Gestational Diabetes	Experiences of blood glucose monitoring	The interviews
41	Zadeh, N. N. et al	Case-control study	100	Gestational Diabetes	Depression and anxiety	GHQ
42	Zulfiqar, Tehzeeb et al	Qualitative Study	23	History of a gestational diabetes	The barriers and facilitators to a healthy lifestyle	The interviews
43	Katon, J. G. et al.	Cross-sectional study	2398	Gestational Diabetes	Antenatal depression	PHQ-9
44	Wilson, C. A.	Cohort study	12,239	Gestational Diabetes	1) Antenatal mental health disorders	medical records
45	Larrabure-Torrealva, G. T.	Cross-sectional Study	1300	Depression	Gestational Diabetes	PHQ-9
46	Beka,Q et al	Retrospective Cohort Study	253,911	Depression	Gestational Diabetes	Diagnosis record

### 3.1 Meta-analysis

Of the 21 studies that were eligible, 12 were used in the first meta-analysis to demonstrate the prevalence of MH outcomes among women with GDM. The second meta-analysis reported the prevalence of GDM among BAME women with pre-existing MH disorders using the remaining 6 studies. Key characteristics of the studies associated with both meta-analyses are shown in Tables 3, 4, 5 and 6.

**Table 3:** indicates 12 studies selected for meta-analysis demonstrating the gestational diabetes sequalae with mental health (Depression).

Study ID	Author	Study type	Sample size	Exposure	Outcome	Outcome assessment	Type of OR and covariates
1	Abdollahi, F et al	Longitudinal Cohort Study	1449	Gestational Diabetes	Post-partum depression	EPDS≥12	Both unadjusted and adjusted OR (covariates are not specified)
8	Byrn, Mary et al	Cross-sectional Study	135	Gestational Diabetes	Depression	EPDS≥12	Adjusted OR (covariates are age, income, marital status, body mass index, and gravida
10	Catherine, K. I. M. et al	Cross-sectional Study	7065	Gestational Diabetes	Poor health and mental distress.	NHIS six item Non-Specific Distress Battery	Both unadjusted and adjusted OR are provided (covarates are demographic factors, BMI and mental health distress)
13(a)	Crowther, C. A. et al	Randomised Clinical Trial	1000	dietary advice, blood glucose monitoring, and insulin therapy	The effect of the intervention on depression	EPDS≥12	Adjusted OR (covariates are maternal age, race or ethnic group, and parity)
30	Huang T et al	Cross sectional Study	1686	GDM	Postpartum depression	EPDS>13	Both unadjusted and adjusted OR (covariates are age, race/ethnicity, education, nativity, parity, marital status, household income, pre-pregnancy BMI and pre-pregnancy physical activity)
34	Katon, J. G et al	Cross-sectional study	2398	GDM	Antenatal depression	PHQ-9	Adjusted OR are provided (covariates are maternal age, marital status, ethnicity, education, one or more other chronic medical condition, prior pregnancy, gestational week prior pregnancy complication)
35	Kim C et al	Case-control Study	1445	Gestational Diabetes	Depression	CES-*D*>10	Both unadjusted and adjusted OR are provided (covariates are age, race, education, prepregnancy weight, prepregnancy exercise level, parity, and prior history of PIH)
36	Kozhimannil, K.B et al	Retrospective Cohort Study	11,024	Gestational diabetes, not taking insulin	Depression	Depression or a prescription drugs	Both unadjusted and adjusted OR are provided (covariates age, race, year of delivery, preterm birth, cesarean delivery)
41	Liu, C. H. et al	Cohort Study	3738	Gestational Diabetes	Postpartum Depression	PRAMS	Adjusted OR are provided (covariates are ethnicity, other sociodemographic factors, stressors, and discussion of mood with provider)
42	Mak, J. K. L. et al.*	Cohort Study	1449	Gestational Diabetes	Depression	EPDS	Adjusted OR are provided (covariates are age, pre-pregnancy BMI, employment status, admission to neonatal intensive care unit and antenatal EPDS score)
63	Walmer, R. et al	Case-control Study	18,109	GDM	mental health disorders	electronic medical records	Adjusted OR are provided (covariates are age, preeclampsia, and preterm birth, marital status, years of education, baby gender, mode of delivery, primary language spoken, number of fetuses, other labor complications, systolic blood pressure, parity, body mass index, weight gain, breast feeding at discharge, and length of follow-up)
65(a)	Wilson, C. A. et al	Cohort Study	12,239	GDM	1) Antenatal mental health disorders	Medical records.	Adjusted OR are provided (covariates are maternal age, education, ethnicity, multiple pregnancy, obstetric complications, preconception CMD, maternal smoking and pre-pregnancy BMI)

*This study actually reported the results at Months 1 and 3 respectively after giving birth, we treated it as a longitudinal cohort study.

**Table 4:** Demonstrates characteristics of the studies included within the Meta-analysis evaluating GDM among women with a depression diagnosis.

Study ID	Author	Study type	Sample size	Exposure	Outcome	Outcome assessment	Type of OR and covariates
7	Bowers, K. et al	Cohort Study	121,260	Depression	Gestational Diabetes	Medical records.	Both unadjusted and adjusted OR (covariates are pre-pregnancy, BMI, gestational weight gain)
14	Dahlen, H. G. et al	Cohort Study	3092	Depression.	Gestational Diabetes	Clinical data from first antenatal visit, through to discharge of mother and baby from the hospital.	Both unadjusted and adjusted OR (covariates are smoking, primip, age, BMI,born in Australia)
25(a)	Hinkle, S. N. et al	Longitudinal Study	2477	Depression in the first and second trimesters	Gestational diabetes	medical record	Both unadjusted and adjusted OR (covariates are age, race, education,, marital status and pre-pregnancy BMI)
39	Larrabure-Torrealva, G. T. et al	Cross sectional Study	1300	Depression	Gestational diabetes	OGTT	Both unadjusted and adjusted OR (covariate are age and family history of diabetes mellitus among first degree-relatives)
64	Wilson, B. L. et al	Correlation study	3655	Depression, stress and physical abuse	Gestational Diabetes	PRAMS self-report	Only adjusted OR (covariates are Race, age, and BMI)
65(b)	Wilson, C. A. et al	Cohort Study	12,239	Preconception mental health disorders	Gestational Diabetes Diagnosis	glucose	Both unadjusted and adjusted OR (covariates are maternal age, education, ethnicity and obstetric complications of preeclampsia, gestational hypertension)

**Table 5:** Demonstrates characteristics of the studies included within the meta-analysis evaluating anxiety among women with GDM.

Study ID	Author	Study type	Sample size	Exposure	Outcome	Outcome assessment
4	Beka, Q. et al	Cohort Study	326,273	Gestational Diabetes	2) Mental illness postpartum	Medical record
5	Beka, Q. et al	Retrospective Cohort Study	253,911	Gestational Diabetes	At least one hospitalization, outpatient visit, or physician claim for a mood or anxiety disorder in any diagnosis field in the 2 years prior to pregnancy	Alberta Perinatal Health Program.

**Table 6:** Demonstrates characteristics of the studies included within the meta- analysis evaluating stress among women with GDM.

Study ID	Author	Study type	Sample size	Exposure	Outcome	Outcome assessment
2	Ahmed, Anwar E et al	Cross-sectional Study	438	Gestational Diabetes	Stress	PSS≥20
62	Silveira, M. L. et al	Prospective cohort study	1308	Gestational Diabetes	Perceived stress	plasma glucose, OGTT

**Fig. 3:**
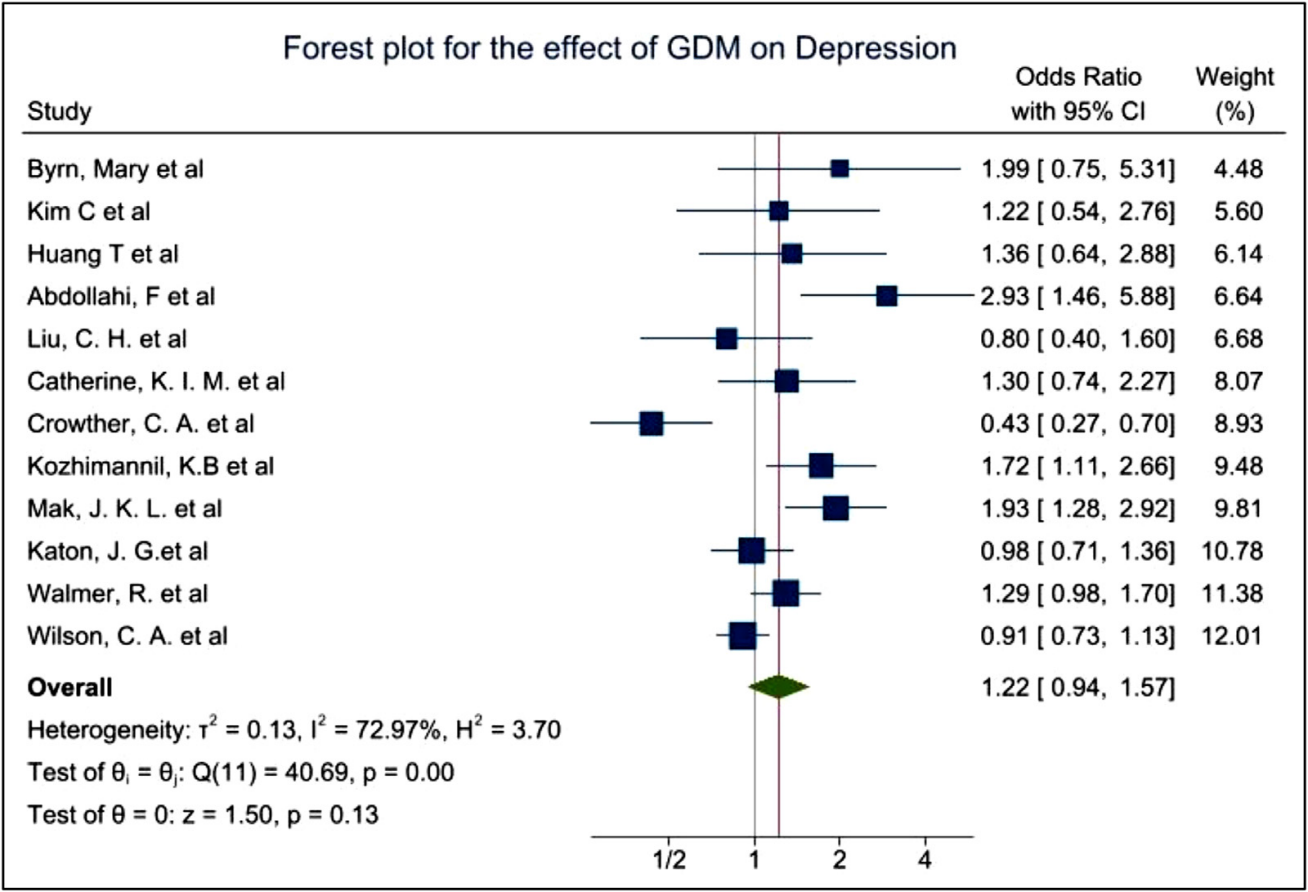
Forest plot showing the prevalence of Depression among GDM women.

**Fig. 4:**
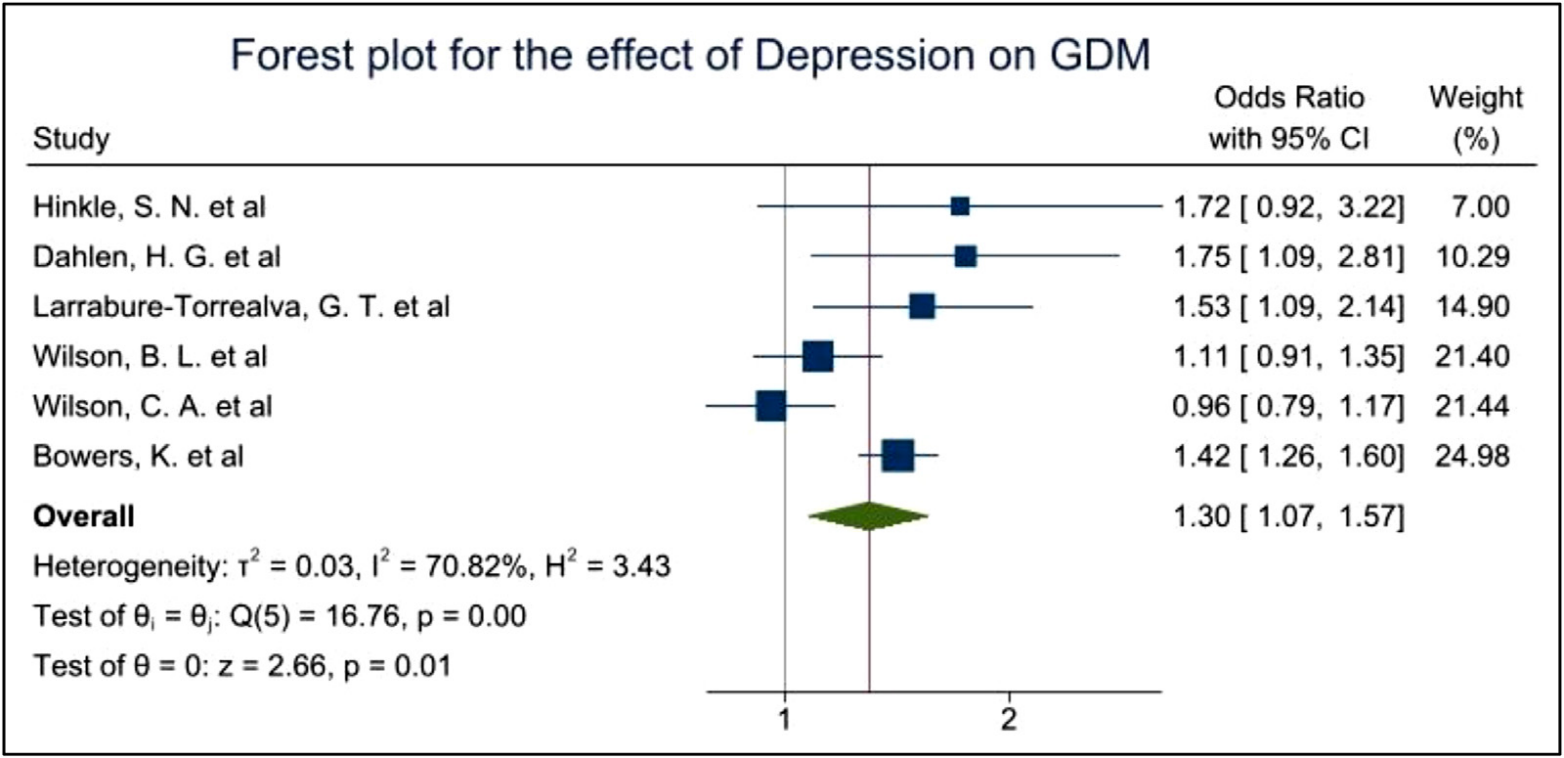
Forest plot showing the prevalence of GDM among women with a diagnosis of Depression.

All studies included in the meta-analyses reported adjusted OR (aOR) and crude OR, as indicated in Tables 1a, 1c and 1d. OR and aOR were used as the pooled estimator to compare the bidirectionality demonstrated by the meta-analyses. Some studies provided more than one aOR. Study 11 for example, used 2 types of aORs, which resulted in a contradictory conclusion and was thus, removed due to quality issues. Mak and colleagues [21] used an aOR for age in particular appeared higher (OR=1.45, 95%CI=(1.15,1.82) and an aOR for covariates such as age, education, preeclampsia, preterm birth, marital status, baby gender, mode of delivery, language spoken and BMI where the aOR was lower (OR1.29 with 95%CI of OR 1.02–1.7,). Therefore, the lower aOR was used for the meta-analysis to reduce biases due to other factors. In some studies, OR of pregnancy associated depression and postpartum depression (PPD) were both included without a clear separation between possible symptomatologies and potential diagnoses, thereby the original data collection and reporting lacks adequate scientific rigor. Due to this, OR of PPD was used to focus on the long-term impact of GDM on women. The results of the two meta-analysis are graphically displayed with forest plots shown in Figs. 3 and 4.

The pooled OR of 1.22 with 95%CI of 0.94–1.57 and p-values of 0.13 indicated a non-significant evidence for the increased risk of depression among GDM women. I^2^ of 72.97% showed high heterogeneity among the studies due to the differences of the study type, covariates, assessment tools, ethnicities and other factors. (Fig. 3) While the pooled OR of 1.30 with 95% CI of 1.07–1.57 and p-values of 0.01 showed significant evidence for the increased risk of GDM in women with history of depression. I^2^ of 70.82% still showed high heterogeneity among the studies (Fig. 4).

**Fig. 5:**
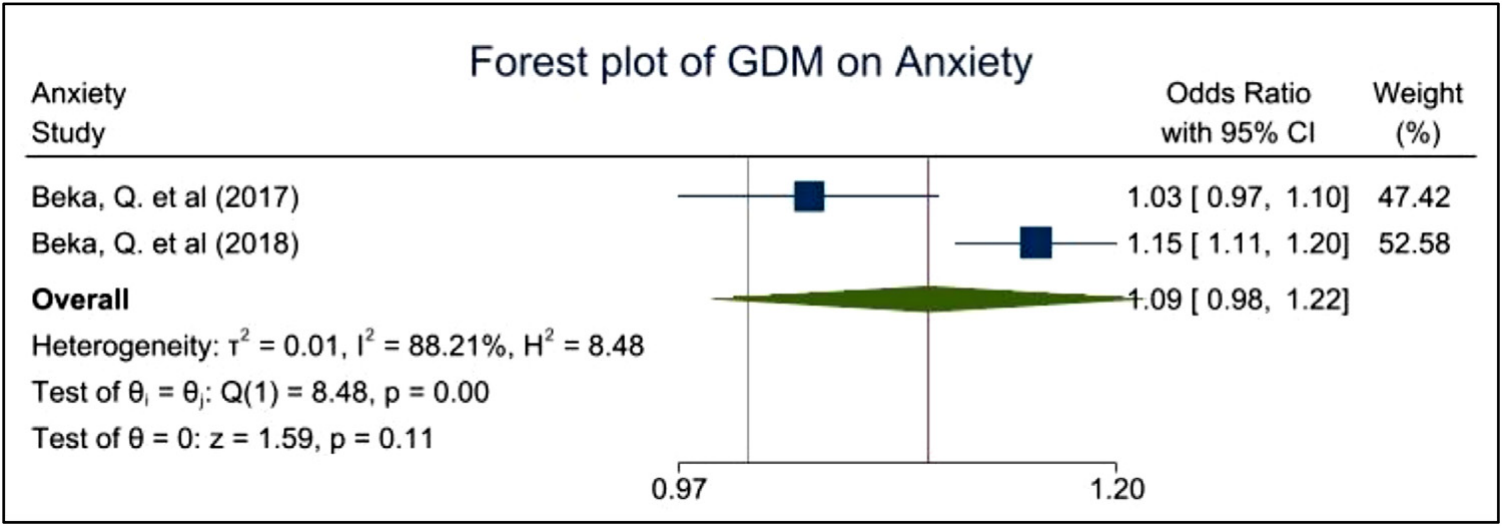
demonstrates the prevalence of anxiety among women with GDM.

Fig. 5 demonstrates the effect of anxiety on women with GDM which appears to be non-significant based on two studies. Studies with GDM among women with an existing MH diagnosis of anxiety reported a pooled aOR of 1.09 with a 95% CI of 0.98–1.22 and a *p*-value of 0.11. This indicates a non-significant evidence of the high prevalence of anxiety among BAME women with GDM. An I^2^ of 88.21% was identified indicating high heterogeneity within the dataset gathered.

**Fig. 6:**
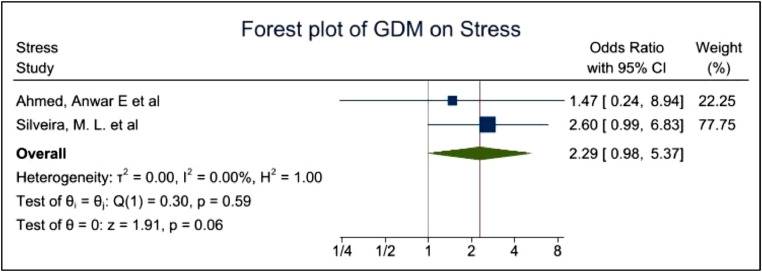
demonstrates the prevalence of stress among GDM women Stress.

Fig. 6 indicates 2 studies reporting the prevalence of stress among women with GDM. These studies report an OR of 2.29, with 95%CI of 0.98–5.37,. P-value of 0.06 showed almost but not significant evidence of the high prevalence of stress among women with GDM and the heterogeneity I^2^ equals to 0%. Therefore, it appears the prevalence of stress among women with GDM is twice as high as those without GDM. However, the sample sizes are minimal to draw any comprehensive conclusions.

### 3.2 Subgroup analysis

To further analysing the sources of heterogeneity, a subgroup analysis was conducted using reported OR, study type and ethnicities.

**Fig. 7:**
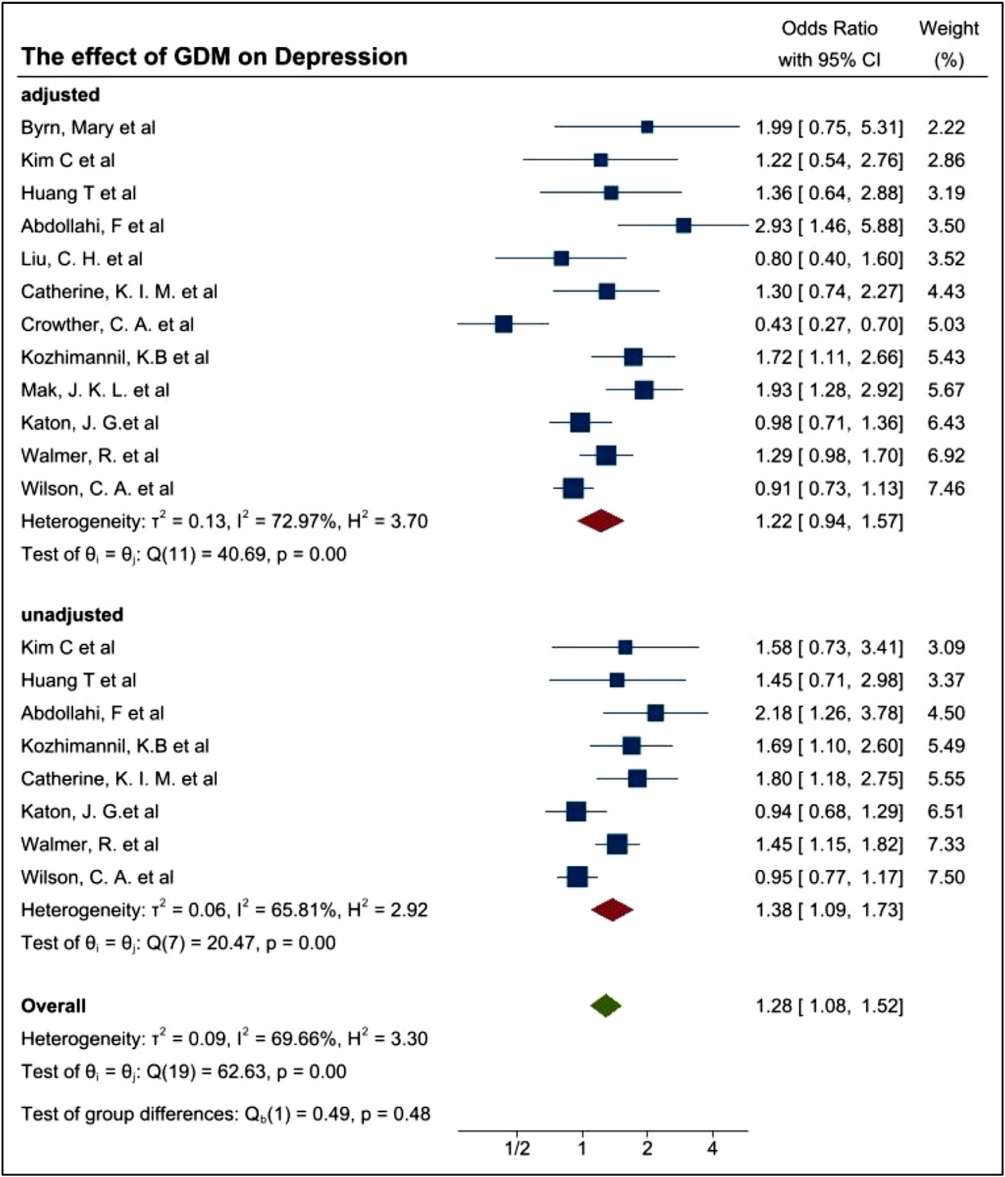
Forest plot demonstrating the prevalence of depression among women with GDM based on unadjusted OR and adjusted OR.

As demonstrated in Table 3, a total of 12 studies were included within the random effects model to evaluate aOR for depression and 8 studies were included to evaluate crude OR (unadjusted OR) for depression. Due to the covariates of adjusted OR were varied in each study, in general, the heterogeneity of adjusted OR between studies will be higher than that of unadjusted OR. Fig. 7 demonstrated the effect of GDM on depression with adjusted and unadjusted OR, respectively.

The pooled OR was 1.38 with a 95%CI of 1.09–1.73 However, the pooled aOR of 1.22 (95% CI of 0.94 −1.57) showed ambiguous evidence for the increased risk of depression in women with GDM. The heterogeneity (I^2^) in the aOR group (72.97%) was higher than the unadjusted group (65.81%), due to the influence of other factors as the unadjusted OR appears to overestimate the variables. Therefore, more emphasis was made to the aOR based outcomes to deduce a conclusion in order to assess the possible source of heterogeneity from the subgroup analysis by way of study type.

**Fig. 8:**
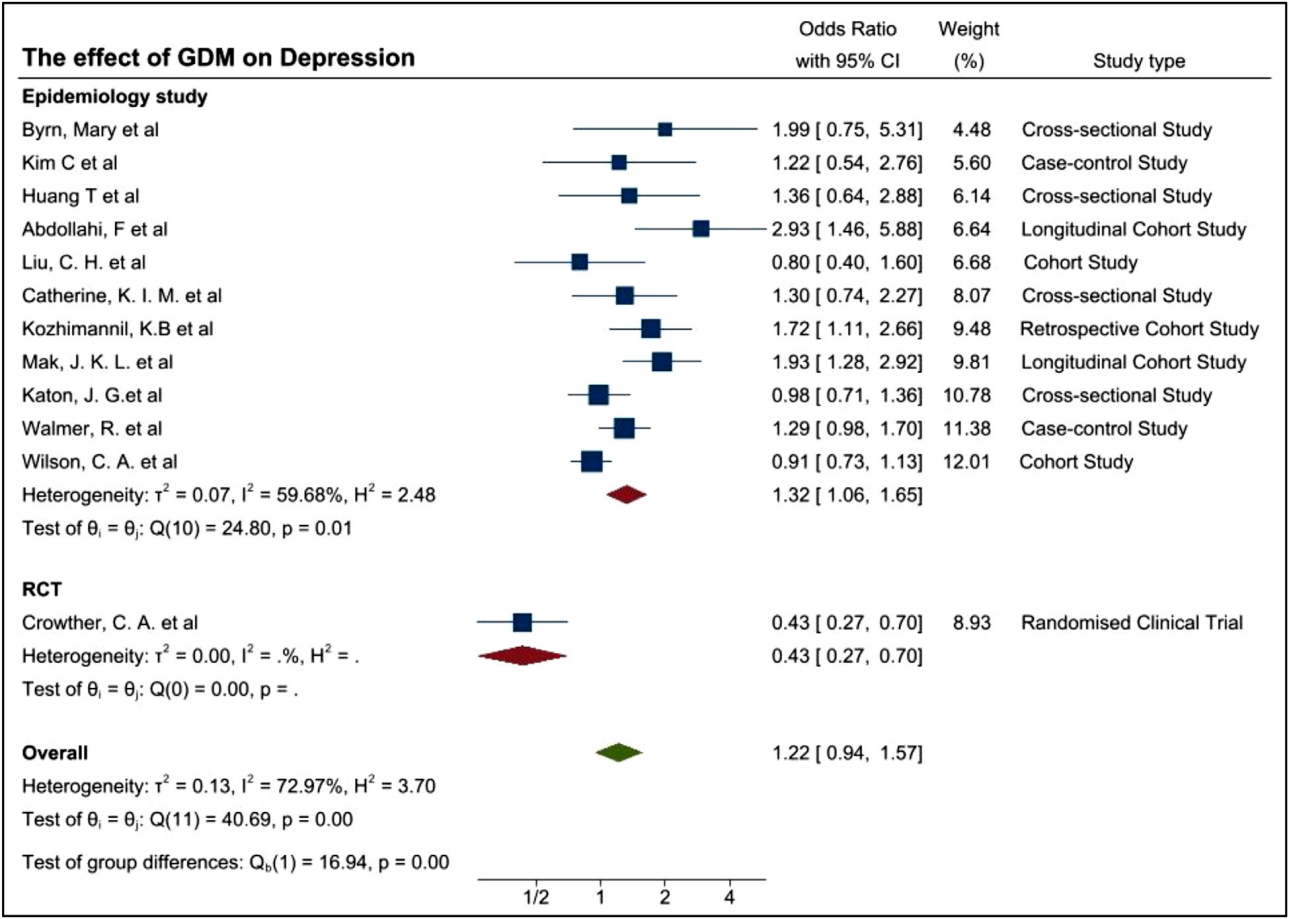

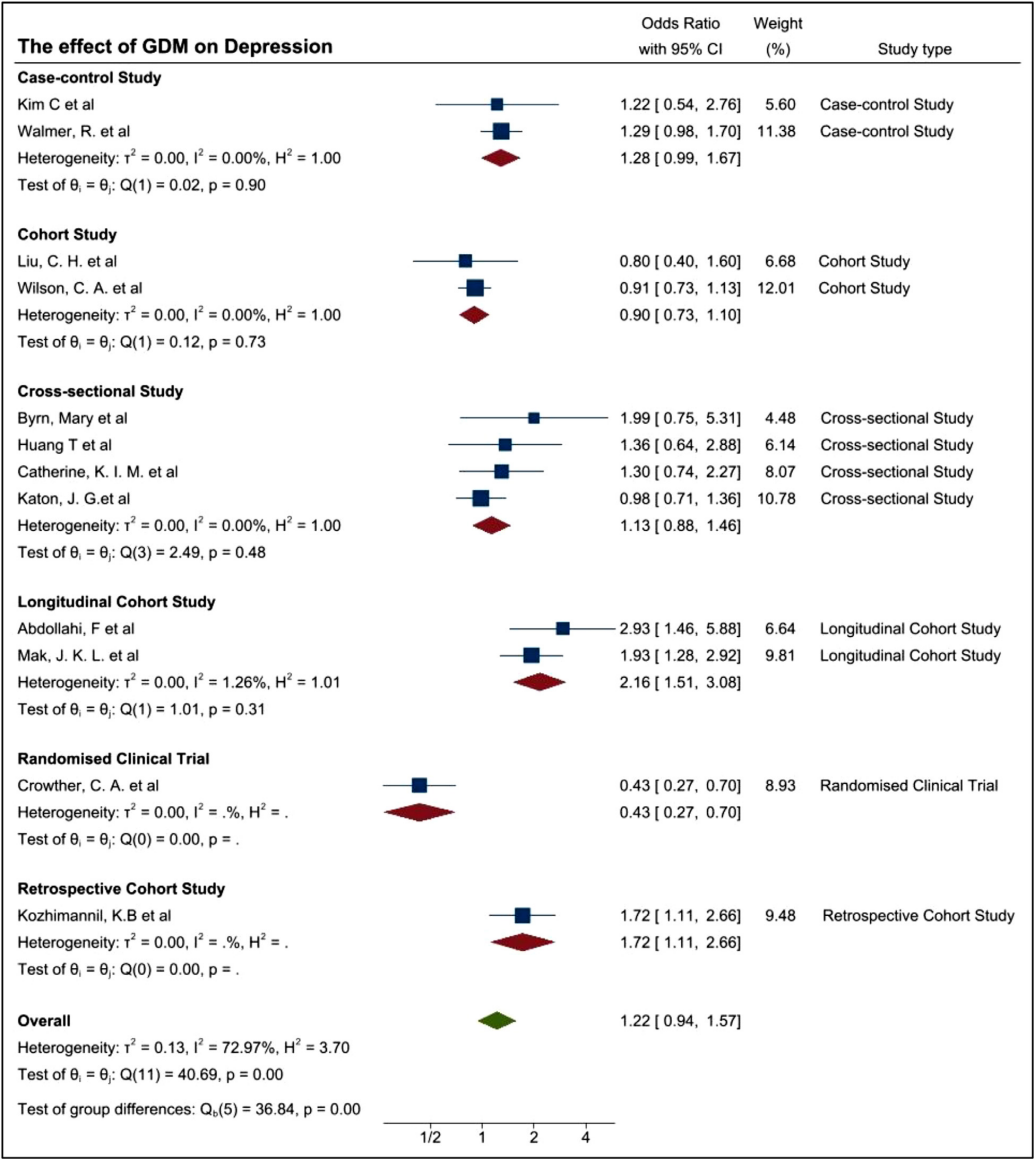
(a) Forest plot demonstrating the prevalence of depression among women with GDM based on study type (b) Forest plot demonstrating the prevalence of depression among women with GDM based on more detailed study type.

To further assess the possible cause of the heterogeneity, study designs were evaluated. In order to assess the study design, initially frequency of the assessments deployed to patients were also considered but this was unclear in some studies and were not unilaterally conducted. Therefore, the study design at a high level was assessed in that, if they were classified into an RCT or non-RCT. The primary non-RCT category is epidemiology based, although these could be further delineated to cross sectional, case controlled, and cohort based. Similarly, some studies were conducted retrospectively and others prospectively. These have been demonstrated in Fig. 8a. Usually, the case-control study could be a clinical trial. However, the 2 case-control studies by Kim, C et al. and Walmer, R. et al. didn't conduct the experiments on two corresponding groups therefore we summarized these two studies as epidemiology study group.

Although I^2^ of 59.68% for the epidemiology group showed a mild heterogeneity, I^2^ is increased to 72.97% (*p*-value 0.00) when we combined both groups, indicating the significant difference between them. The estimates of the OR are 1.32 (95%CI 1.06–1.65), 0.43(95%CI 0.27–0.70), 1.22 (95%CI 0.94–1.57) respectively for epidemiology group, RCT group and the pooled data, meaning there may be some association between GDM and MH disorders but having no strong evidence. Further studies are required.

The pooled aOR, 95%CI and I^2^ in each subgroup were listed in Table 1c. The longitudinal cohort study and retrospective cohort study showed a significantly higher prevalence of depression among women with GDM. Cross-sectional study, case-control study, cohort study came to a conclusion that the ambiguous evidence of high prevalence of depression could be found among women with GDM. Randomised clinical trial showed a significant lower prevalence of depression among women with GDM. There's little heterogeneity in each subgroup. We could find the heterogeneity in meta-analysis was likely due to the differences of study type (study design, assessment timepoint, selection of control group etc.). Because the number of each study type is too small, it's hard for us to get an accurate conclusion, while what this rough conclusion could give us was we should try to separate and merge the conclusions according to the study type, otherwise our conclusion may be difficult to achieve statistical significance.

**Fig. 9:**
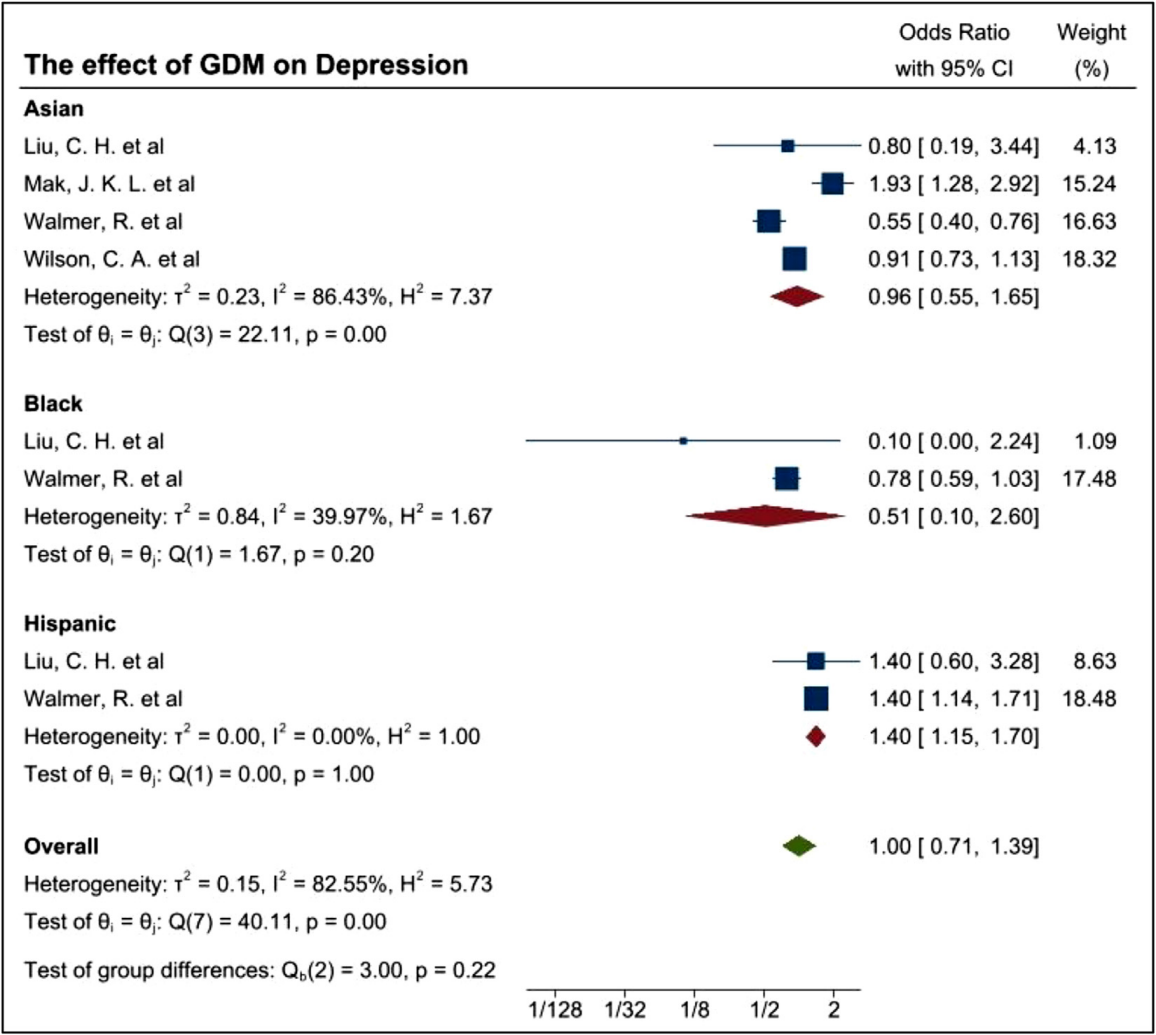
Forest plot demonstrating the prevalence of mental health outcomes among women with GDM based on ethnicity.

A subgroup analysis on ethnicity was conducted to align with the scope of this study. Fourteen studies (Table 7) used within the meta-analysis was used to determine the association of ethnicity and GDM. Of the 14 studies, seven studies comprised of women from Iran and Saudi Arabia decent whilst 7 consisted of Hispanic women. However, following a quality assessment, only four studies were used within the subgroup analysis as demonstrated in Fig. 9.

**Table 7:** Demonstrate the studies selected for subgroup analysis.

Study ID	Author	Study type	Exposure	Outcome	Outcome assessment
41	Liu, C. H. et al	Cohort Study	Gestational Diabetes	Postpartum Depression	Pregnancy Risk Assessment Monitoring System survey
42	Mak, J. K. L. et al	Cohort Study	Gestational Diabetes	Depression	EPDS
63	Walmer, R. et al	Case-control Study	Gestational Diabetes	Mental health disorders	Electronic medical records
65(a)	Wilson, C. A. et al	Cohort study	Gestational Diabetes	1) Antenatal mental health disorders	medical records

**Table 8:** Indicates Predefined Clinical Variables.

Anxiety scores
Low mood
Depression
Treatment resistant depression
Sleeping disturbances
Diet
PTSD
Suicide
Risk factors
Exercise
Risk and risk perception

The analysis indicates Hispanic women with GDM were at greater risk of MH conditions. The aOR analysis of these studies indicated Hispanic women with GDM were at greater risk of MH outcomes with an aOR of 1.4 and 95% CI of 1.15–1.7 which equates to an I^2^ of 0%. For Asian and Black women, the aOR was 0.96 with a 95%CI of 0.55–1.65 and 0.51 with 95%CI of 0.1–2.6, respectively. An I^2^ of 86.43% and 39.97% were evaluated, respectively among the Asian and Black women. Both groups had ambiguous evidence in regard to the high prevalence of identified depression. High heterogeneity identified may due to the diversity of ethnicity, race as well as the differences in the sample sizes and MH assessments provided.

### 3.3 Publication bias

**Fig. 10:**
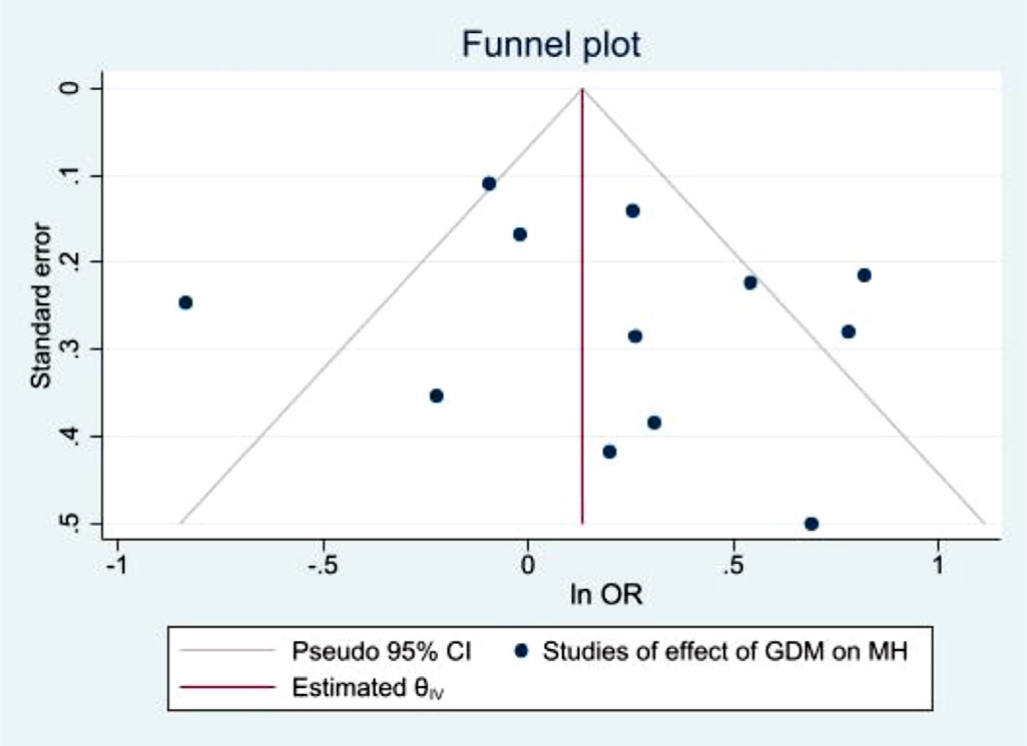
Funnel plot with pseudo 95% confidence limits for studies included in the meta-analysis of prevalence depression among women with GDM.

**Fig. 11:**
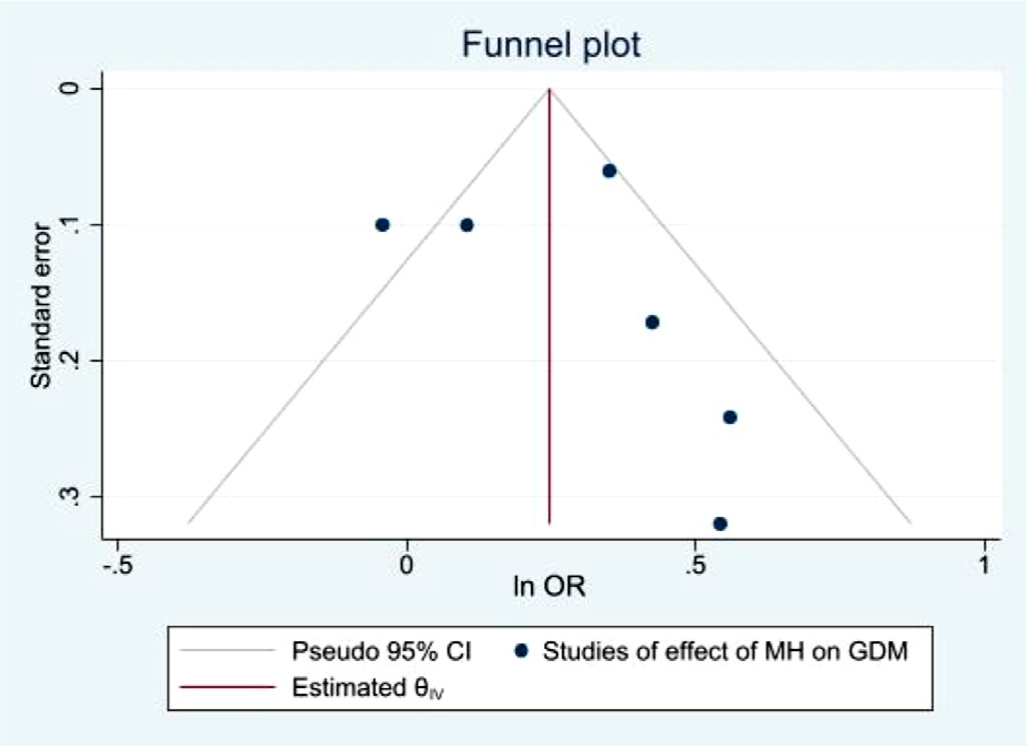
Funnel plot with pseudo 95% confidence limits for studies included in the meta-analysis of prevalence GDM among women with a diagnosis of depression.

**Fig. 12:**
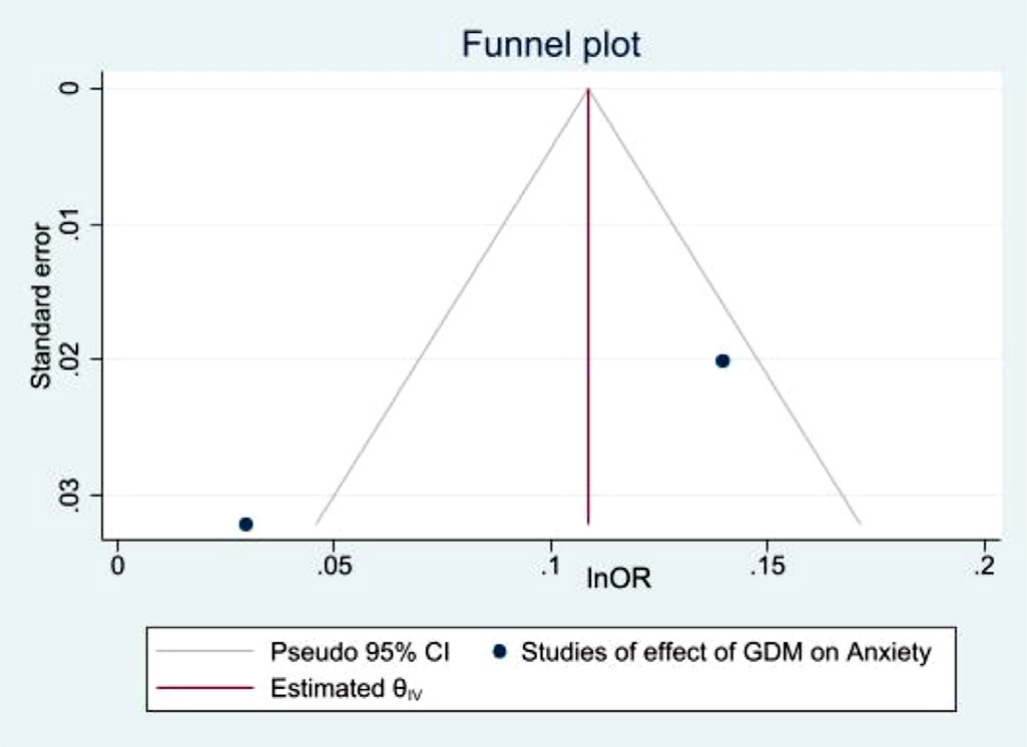
Funnel plot with pseudo 95% confidence limits for studies included in the meta-analysis of prevalence anxiety among women with GDM.

**Fig. 13:**
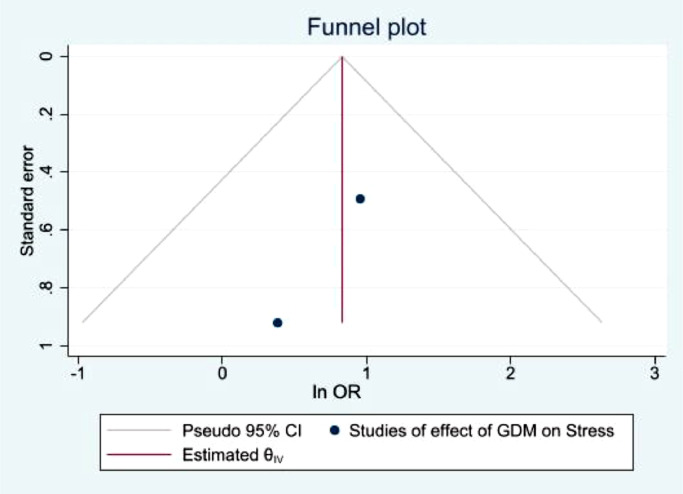
Funnel plot with pseudo 95% confidence limits for studies included in the meta-analysis of prevalence stress among women with GDM.

**Fig. 14:**
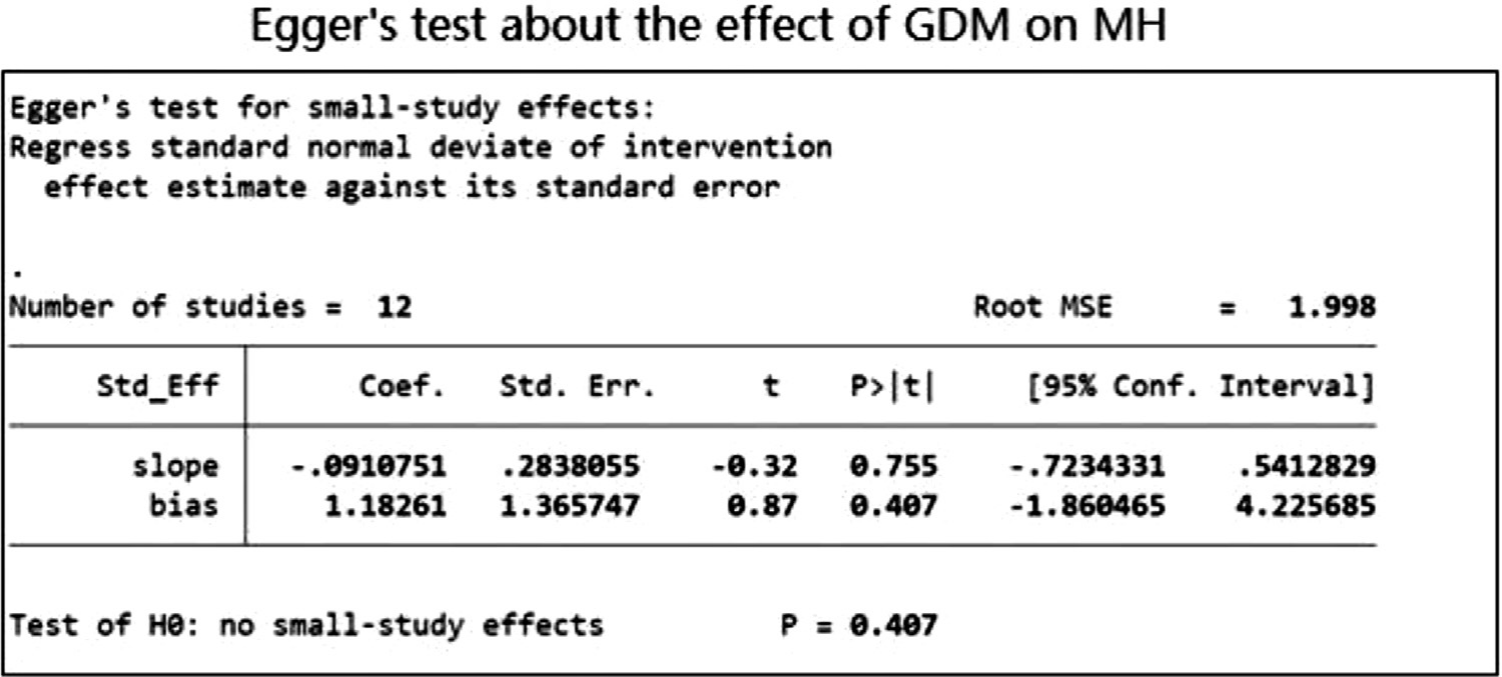
Egger Test for studies demonstrating the MH impact among GDM women.

**Fig. 15:**
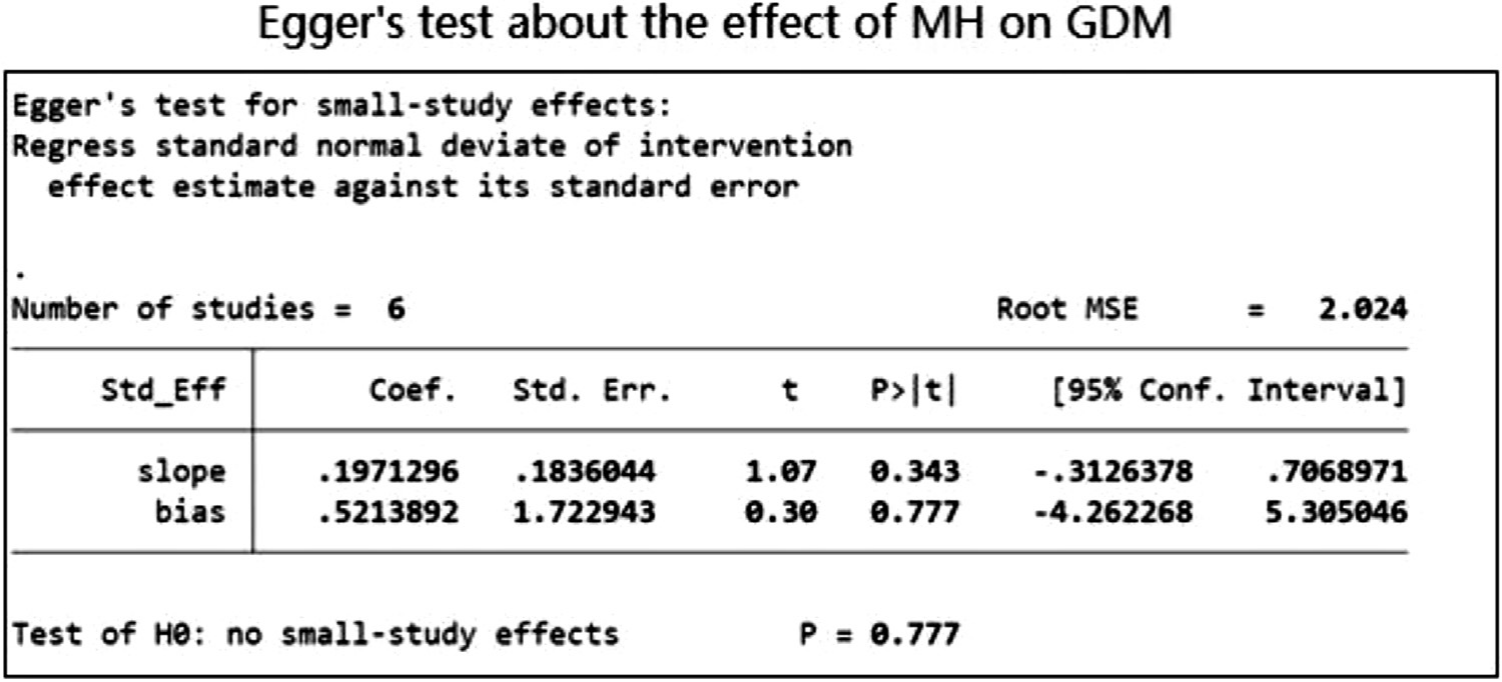
Egger Test for studies demonstrating the prevalence of GDM among women with a pre-existing mental illness.

The funnel plots illustrated in Figs. 10,11,12,13, demonstrate a clear indication of statistically evaluated minimal publication bias. The *p*-values of the Egger's tests (Figs. 14 and 15) for the meta-analysis reporting depression among women with GDM, was 0.407. The meta-analysis assessing the prevalence of GDM in women with pre-existing MH conditions reported a *p*-value of 0.777 indicating a lack of an effect size. As a result of this, publication bias cannot be reached. This is indicative that women with a pre-existing diagnosis of depression may have a higher risk of GDM. Women suffering from GDM are 22% (excess risk in terms of OR) more likely to suffer from depression than those without GDM based on the evidence of this study.

## 4 Discussion

In this systematic review and meta-analysis, a prevalence of GDM and MH outcome of depression, anxiety, stress and psychological distress were identified with key themes including clinical variables demonstrated in Table 9. The evidence gathered also demonstrated a bidirectional relationship between GDM and MH. The meta-analysis indicated a significant relationship between GDM and PND in particular, although some studies also reported other MH outcomes such as psychological distress.

**Table 9:** indicates themes identified within the systematic review.

Themes	Population Group
	Gestational Diabetes and Mental Health sequalae amongst Black, Asian and Ethnic Minority Women
Depression	++++++++++++++
Post-partum depression	+++++++
Gestational diabetes	++++
Anxiety	+++
Stress	++
Quality of Life	++
Experiences related to gestational diabetes	++
Attitudes and health behaviours	+
Beliefs about health	+
Beliefs about illness	+
Perinatal Depression	+
Postnatal depression	+
Experiences of GDM	
Lifestyle Change	+
Support and environmental influences related to gestational diabetes	+
Experiences of bloody glucose monitoring	+
Barriers and facilitators to a healthy lifestyle	+
Antenatal depression	+
Antenatal mental health disorders	+

The levels of evidence differ from the meta-analysis and the systematic review which, demonstrates GDM increased the risk of maternal MH conditions as reported by 16 studies, although, they did not specify the prevalence of depression or anxiety to each ethnic group of the population. Four studies suggested that GDM heightened the risk of MH conditions, yet they did not analyse these effects according to ethnicity [7,47,54,58]. On the contrary, Nicklas and colleagues reported that ethnicity did not alter the risk of PPD [9], following adjustments for demographic variables. 2 studies reported that GDM did not alter the risk of PND in Hispanic [55] and Iranian [65] women. Another large cohort study reported that African American women with GDM had a lower risk of PPD in comparison with Caucasians [21]. Dahlen and colleagues [52] stated that participants from India or Pakistan did not report any MH disorders during pregnancy. Eleven studies reported a lack of association between GDM and the development of maternal MH condition. Six studies stated that GDM did not significantly increase the risk of mood disorders, although this finding remain non-specific to the BAME community [49,53,[61], [62], [63],^78^]. Since mood disorders encompass a wide range of conditions, this would preclude the assessment of the prevalence of the specific MH sequalae of GDM. It remains to be seen if this is due to stigmatisation of MH disorders within the BAME community has influenced any possible under-reporting as suggested by Gary and colleagues [16,85].

Of the 46 studies, 2 reported that MH conditions could be more prevalent amongst immigrants and Black women with GDM compared with control groups [18,30]. Furthermore, eight studies that were conducted in countries where the native majority are non-Caucasians found that predisposition to GDM could develop MH conditions [21,48,56,64,66,79,83,84].

Of the 26 studies that reported psychological experiences of women with GDM [12,[22], [23], [24], [25], [26], [27], [28], [29], [30], [31], [32], [33], [34], [35], [36], [37], [38], [39], [40], [41], [42], [43], [44], [45], [46]], there appears to be psychological distress associated with concerns related to the baby [23], blood glucose monitoring [45] and administering insulin injections [39]. Whilst these studies highlighted the emotional burden of GDM, the self-reported, subjective descriptions of the psychological distress experienced by these women, a clinical diagnosis remained absent. It remains unclear if these patients were unable to access healthcare services, although Kim and colleagues [80] indicated that women with GDM were themselves, unlikely to approach MH providers.

Nineteen studies reported from non-American countries where the majority of the study population comprised of non-Caucasian groups. This is particularly important given the impact of culture on mental illness and its relationship with psychopathology, psychosocial behaviours, treatment interventions and overall outcomes. There are cultural differences and norms in perinatal care and parenting styles within BAME groups that could be considered as potential stressors impacting on the overall health of this group. Trends in mass migration, presumably could play a role in a healthcare system's ability to support the clinical needs of BAME populations with GDM and/or MH disorders. A study conducted by Hjelm and colleagues [31] reported that although immigrant women with GDM experienced higher levels of anxiety compared to native study participants, findings varied according to acculturation status. Migrant women experiencing language barriers reported frustration accessing healthcare [32] and some BAME women did not feel satisfied with care [28]. Some women deemed dietary and lifestyle advice unacceptable and resorted to traditional herbal remedies [26] or spiritual measures [27] to manage the condition. Due to the small sample sizes demonstrated within these studies, the generalisability of the findings were challenging to quantify.

In the United Kingdom, the National Institute of Clinical Excellence (NICE) recommends that women from ethnic groups with a high prevalence of diabetes should be invited for testing [70]. For some women, this may be an unexpected diagnosis, or one associated with negative connotations and could be considered as *perceived stigma*. International Diabetes Federation (IDF) provides practical guidance on the impact of fasting on physical and mental wellbeing and management of diabetes in vulnerable groups and specifically in pregnant women and recommendations for safely participating in Ramadan [87]. The additional concerns for their health as well as those of their unborn child may lead to further psychological distress. This could exacerbate underlying health fears associated with race and the impact that it has on their clinical care [71].

Impact of stigma and shame of mental illness within BAME communities should be understood in the context of the individual, family and the respective minority group as this subject matter often remains a “*taboo*”[72,73]. This may influence positive reinforcement behaviours, prompting a need to seek clinical assistance instead of using traditional remedies, which is common within BAME populations [72,73]. Rathod and colleagues [74] and Phiri et al. [73] demonstrated stigma of mental illness could be worse among South Asian Muslim women in particular, for example due to fear of being labelled as “*mad*”. African Caribbean women on the other hand believe resilience could often impact insight into illness in general and, especially denial of any mental illness [73,74]. These beliefs could lead to poorer MH during the antenatal and postnatal period, particularly, if GDM has led to unexpected interventions such as induction of labour by way of emergency caesarean section. Pregnancy and puerperium are a particularly sensitive period for a woman and the additional burden of a now high-risk pregnancy may further exacerbate any underlying mental illness. stigmatization among ethnic minority communities may inhibit BAME women from seeking MH support [60], particularly if there are concerns around possible detention under the MH Act in the UK, for treatment [75,76]. For example, it has been reported that Bangladeshi communities perceive depression as a sign of weakness [81]. Another issue appears to be the lack of awareness of perinatal services in some women from BAME groups and language barriers including differing attributions to mental illness, contribute to known barriers and access issues. This important issue highlights not only the challenge of recognizing MH disorders amongst BAME women with GDM but cultural awareness and sensitivities that could aid with improving engagement with these communities [82].

There is evidence to suggest healthcare providers recognised that anxiety reported by GDM patients [56] and healthcare professionals understood the long-term implications of the condition [56]. One study reported that healthcare professionals were anxious to optimize GDM pregnancy outcomes [41]. This caused some women to feel overly scrutinised thus, reporting an exacerbation of perceived stress. One study acknowledged that although women commonly feel anxiety associated with GDM, midwives may find it challenging to recognize this emotional response [57]. A common theme discussed in the studies were that healthcare providers may not provide culturally appropriate advice to BAME women with GDM. Healthcare advice regarding diet and exercise may contradict traditional beliefs about pregnancy [53]. This conflict increased anxiety in some women who found new dietary recommendations challenging in terms of adherence [33].

**Fig. 2:**
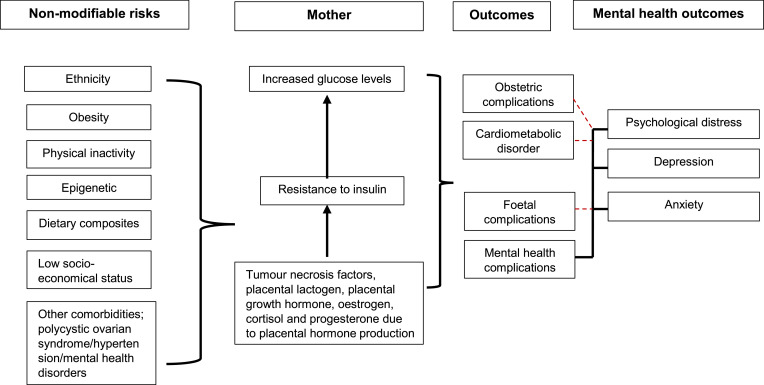
a Gestational Diabetes Mellitus and Mental Health *‘Causation Tree’*.

The findings of this review may appear out of keeping with information that is known about the aetiology of mental illness being increased in certain BAME populations [75,76]. This may be attributable to differences in reporting and, diagnostic tools used across the different studies. The prevalence of MH outcomes may have been under-reported due to the inconsistent use of screening tools across many studies. The most utilised tool to diagnose depressive symptomatology for example was the Edinburgh Postnatal Depression Score (EDPS). Twelve studies used EDPS although, with a range of threshold scores to identify depression between 7 [77] and 13 [52,53]. This striking difference would naturally affect the reported prevalence of MH outcomes. MH assessments conducted among GDM patients within the general population varies considerably and remain non-specific to BAME patients. The heterogeneity between adjusted studies is high although statistically, this could be attributed to the different MH assessment criteria, study design and sample sizes used. Common MH diagnostic assessments used were EPDS, PSS, NHIS, CES-D and PHQ-9 (S Fig. 2). These measures are validated with good reliability for many diseases. Although, there is a need to validate these measures and adapt them to be culturally sensitive when used among BAME populations. The use of medical records to identify MH outcomes reported by patients was insufficient to demonstrate the care offered to these patients. However, the cut-off scores to measure severity of depression for example differ across all the studies identified, thus, there is evidence to indicate a high degree of subjectivity. Based on the funnel plots (Fig. 10, Fig. 11), it is evident there is publication bias. Some studies demonstrate an univariate regression despite a lack of significant evidence between GDM and postpartum depression (PPD) for example, thus, OR and 95% CI were unreported. This may also lead to a publication bias.

PPD, depression and anxiety were the key MH themes identified within this review. Additionally, diagnostic criteria for GDM patients reporting MH symptomatologies lacked a clinical diagnosis. This is in line with 5 studies that identified and reported mood disorders. However, a mood disorder theme is a broad classification, with limited value as a clinical outcome measure thus, precluded determination of their scientific merit. The prevalence of mood disorders could also be considered as underreported if study participants were not followed up for a period of time. Some research suggests that women may experience PPD up to a year following birth [7]. Only two studies measured depressive symptoms for this length of time [40]. Whilst it may be unfeasible to follow-up women for a year post-partum, there is a possibility MH disorders amongst women with GDM may have been under-recognised in many studies.

The relationship between GDM and depression itself is complex. A history of pre-pregnancy MH disorders was found to increase the risk of GDM in 8 studies [7,[50], [51], [52], [53],^62^,^78^,^79^]. Larrabure-Torrealva and colleagues [79] found that pre-pregnancy depression was significantly associated with GDM in Hispanic women. A further six studies used data from medical records to diagnose depression or anxiety [18,49,51,59,60,62] and a study by Kim et al. [80] considered contact with a MH provider as an outcome. Therefore, the reporting variability associated within these studies further introduces rate limiting factors to better clinically treat these women.

Future studies should use standardised diagnostic measures for MH and GDM, to allow better generalisability and reproducibility of the data. The impact of the cultural norms and social stressors as well as the effect that these have on mental well-being could be better encapsulated by incorporating more qualitative and quantitative methodology. The disintegration between obstetric and general practice is an important area for healthcare providers to address to ensure joined up services are able to provide a holistic approach to clinical care. Whilst, it is well recognised that women with GDM need postnatal follow-up in view of the risk of developing T2DM in later life, the impact of psychological health and the role that this may have on women from BAME groups is often overlooked and hence, needs to be acknowledged based on the findings of this review. Furthermore, this proposition is justified by the existing knowledge in mainstream MH research, that marginalised communities are disproportionately represented among sufferers of MH illnesses.

Women with psychiatric disorders may have severe consequences during their pregnancy as demonstrated by Damé and colleagues [84] where 8.3% of participants with GDM had experienced thoughts of self-harm. Untreated depression in pregnancy has been associated with adverse pregnancy outcomes for both mother and child [18,86]. However, the cross-sectional studies included small sample sizes therefore, the generalisability of these findings was limited. Recruitment of BAME patients were sub-optimal across many studies, and sub-group analysis based on ethnicity or race were seldomly conducted.

In Asian groups in particular, it could be said that the effect of GDM and increased prevalence observed in MH outcomes were insignificant. Within this group, I^2^ (86.43%) showed a strong heterogeneity. The source of heterogeneity is non-specific to a race as Liu et al. [57] and Walmer et al. [59] focused on the Asian/Pacific Islander women from the USA whilst, Mak et al. [21] included only Chinese women. Effective conclusions will only be sought when racial subgroups are evaluated, and data is more homogenous. Although, when discussing a sequalae, heterogeneity would be challenging to manage. On the other hand, aOR for Black women demonstrating MH outcomes with a GDM diagnosis was 0.51 with a 95%CI range between 0.10 and 2.60 with little heterogeneity (I^2^ =39.97) as well as a *p*-value of 0.20. This indicates the presence of moderate evidence of heterogeneity. Interestingly, the studies of Liu and colleagues [57] as well as Walmer et al. [59] concluded that black women with GDM were less likely to have MH disorders, with the pooled OR showing too wider range [95%CI: 0.10, 2.60] to obtain significant evidence.

Based on the lack of BAME women identified within GDM research and/or MH research associated with women's health in general, there appears to be an issue of under-representation, which raises concerns around the viability of the clinical management aspects offered to these patients. Lapolla and colleagues’ [36] surveyed the perceptions and attitudes of immigrant women with a specific questionnaire in comparison to that used for the general population in Italy, which may raise clinical, scientific and research ethical issues. The non-standardised conduct of such a survey lacks scientific and clinical justification resulting in possible bias in reported outcomes. The use of interpreters to increase participation further raises concerns around the potential variation in terminology used. This approach was furthered by a study conducted by Muhwava and colleagues [39] that used focus groups where a research assistant translated these discussions into two local dialects. Although translations were cross-checked, this introduced a possible risk of bias. In contrast, several other studies excluded women who could not speak English [12,23,26,34,53,69], which could be perceived as possible discrimination. Lack of cultural paradigm-based methodology and incremental procedures to reflect these during the study design process, further purports concern around the translation of research outcomes to clinical practices, where BAME populations are involved. This may have affected the reported prevalence of MH conditions as BAME women with poor English language skills may experience additional psychological strain when seeking antenatal care [85]. A possible solution could be the use of interviewees and other research staff of similar ethnic backgrounds as demonstrated by McCloskey and colleagues [37] that may have encouraged women from diverse ethnic backgrounds to disclose their honest experiences. In contrast, a Danish researcher conducted interviews amongst Tamil women in a study by Nielson et al. [41], where although the researcher may not have identified culturally important factors, their unbiased opinion may have enabled identification of new trends.

The differentiation of reporting symptomatologies against a MH diagnosis was poor amongst all studies included in this review. Therefore, it is not possible to predict the quality of life of the GDM women long-term and if they suffered with any other clinical implications. The studies used a variety of MH assessment tools introducing differences in reporting MH symptomatologies influencing the heterogeneity detected within this systematic review. There appears to be a *sequalae* between MH and GDM (and vice versa) among the BAME population. Further research is required to comprehensively evaluate if there are any mechanistic basis to this relationship, to identify the relevant pathophysiology. Studies were excluded if they discussed quality of life (QoL) but did not refer to specific MH outcomes such as pre or postnatal depression, anxiety, psychosis and generalised mood disorders. The results of several studies indicated that GDM may be closely linked with both MH and QoL. These articles were not included due to the lack of specificity as well as evidence indicating the use of specific MH assessments. Mechanisms to better evaluate this paradigm requires further studies with larger sample sizes and comprehensive study designs. Similar conclusions have been reported by Metelli and colleagues [88] where the use of cross-sectional studies in a meta-analysis could demonstrate high heterogeneity.

Based on the evidence gathered from these meta-analyses and narrative analysis, future research studies should improve their sampling methods to enable better generalisability of their findings. The meta-analysis between GDM and MH indicates a significant impact on the increased risk of PND. MH outcomes such as depression and anxiety were also identified. Depression, anxiety and other psychiatric comorbidities should be further examined within the patients from BAME population where GDM is the primary clinical condition and vice versa. Although, existing literature demonstrates generalised prevalence data, it is evident that limited BAME specific GDM and MH research is available and existing literature demonstrates generalised prevalence data. This could affect global healthcare systems, for example, those in European countries and in Northern America, at a time where migration may alter the population demands from healthcare systems. Furthermore, understanding and comprehensively reporting the disease *sequalae* could support policy makers to develop and implement more equitable clinical services as well as improve patient access frameworks that would advance clinical and patient reported outcomes.

Methodological rigour required for an evidence synthesis that uses multiple analyses methods should be considered based on the clinical condition being examined. A major concern around the use of observational evidence is perceived to be internal validity which include bias and confounding. However, this should be assessed based on the clinical condition and research question of the evidence synthesis as, observational study designs could be a strength to assess epidemiological outcomes such as prevalence. Study designs are equally important to assess as they impact the internal validity of the observation data set, especially to assess the causal relationship between interventions and outcomes. In the presence of limited data, interventions should be reviewed narratively or thematically instead of a meta-analysis as this may introduce bias within the summary effect. Clinical researchers should consider these aspects during the study design process as their work could be used in the context of an evidence synthesis or an independent systematic review in the future. This could introduce both methodological and clinical heterogeneity which would preclude their work from being considered of high scientific quality and limit any meaningful clinical recommendations from being reported.

It is vital to acknowledge that MH symptomatologies and/or disorders could be effectively managed using pharmacological treatments in conjunct with psychological interventions such as Cognitive Behavioural Therapy (CBT) as recommended by the NICE guidelines in the UK. Cultural adaptation will facilitate a better understanding of the suitability of a clinical management regimen for BAME populations, as well as increase patient engagement with healthcare organisation, thus could aid in personalising clinical care using evidence-based approaches. In relation to healthcare professionals, cultural competency training should be recommended when working with diverse populations. Policies and practices could be adopted across multiple healthcare systems and manage even *impromptu* changes in a sustainable manner. Further research on developing culturally sensitive outcome measures for determining GDM and MH in BAME women is warranted.

## Declaration of Competing Interest

PP has received research grant from Novo Nordisk, and other, educational from Queen Mary University of London, other from John Wiley & Sons, other from Otsuka, outside the submitted work. SR reports research funding associated with other studies from Janssen, Otsuka and Lundbeck. DKH reports grants from Wellbeing for women, which has been received for studies external to the ELEMI project. NT reports grants from Wellbeing women and the National Institute of Health Research (NIHR) for studies external to the ELEMI project. This research is based on evidence gathered systematically and have not been influenced by personal expertise. All authors have relevant experiences and expertise for the ELEMI project. All other authors report no conflict of interest.

The views expressed are those of the authors and not necessarily those of the NHS, the National Institute for Health Research, the Department of Health and Social Care or the Academic institutions.

## Appendix Supplementary materials

Image, application 1
