# Supplementary material for: A systematic review and meta-analysis of gestational diabetes mellitus and mental health among BAME populations
Source: eClinicalMedicine. 2021 Jul 14;38:101016. doi: 10.1016/j.eclinm.2021.101016 (PMC8283332; doi:10.1016/j.eclinm.2021.101016)
Supplement: Supplementary file 1 [file mmc1.docx]

| **Mental Health Questionnaires** | **Scale** | **Relevance to BAME population** |
| --- | --- | --- |
| CES-D | 0-3; 0= rarely or none of the time, 1= some or little of the time, 2= moderately or much of the time and 3= most or almost all of the time. | Has been adapted to the Korean version (CES-D-K) this was reliable and valid for Korean (Cho & Kim, 1998) and Armenian population (Demirchyan, Petrosyan & Thompson (2011).  CES-D Cronbach’s α = 0.825 indicative of high internal reliability in Bolivian patients Schantz et al., 2017). In South Africa the 10 item version was validated in the Zulu, Xhosa and Afrikaans population (Baron, Davis & Lund, 2017) (α = 0.69-0.89), and adequate concurrent validity |
| BSI | Items are scored on a 5-point Likert scale, ranging from 0 (“not at all”) to 4 (“extremely”). | Been tested over 400 studies, with non-white sample ranging between 15% in nonclinical adult population, 33% in clinical population , 44% in inpatients and 42% in adolescent non clinical population therefore has reliability and validity |
| EPDS | Scores of 10-12 represent borderline and 0-9 not depressed | Used in Caribbean women with Perinatal depression (Edge et al., 2004) Also validated in Nigerian women (Uwakwe et al 2003) |
| DASS-21 | Respondents rate items on a 0 (‘did not apply to me at all’) to 3 (‘applied to me very much/most of the time). | yes |
| BDI-II | Each item is rated on a 4 point scale ranging from 0-3 and has a total score of 63.  Ranges 0-13- minimal; 14-19 mild; 20-28 moderate and 29-63 severe depressive symptoms | Outpatient sample had 4% (21) African American and Asian American 4% (18) and Hispanic 1%  However, has validity and is widely used in psychiatry |

The centre for epidemiological studies-depression (CES-D) is a 20 item scale used to rate caregiver’s experience of depression symptoms such as poor appetite, insufficient sleep and loneliness over the past week. Responses to these questions range from 0 to 3 where the total score could be 0 to 60. CES-D provides cut off scores which aids with identifying those with a higher risk of clinical depression. Roberts and colleagues (1997) report that CES-D demonstrates a high level of specificity, consistency and sensitivity, therefore, would be useful to determine differences between caregivers and non-caregivers (Pinquart et al 2003). Pinquart and colleagues (2006) also reports CES-D is suitable to determine post-interventional depressive symptoms in non-caregivers. Although, the use CES-D within BAME populations may requires further evaluation given the lack of different factor structures such as cultural paradigms relevant to ethnic caregivers.

The brief symptom inventory (BSI) which includes 53 items use a factor analysis and is a version of the symptom checklist SCL-90 (Franke et al 2017). BSI use a scale structure to measure psychological distress and comorbidities such as cancer, COPD (Coultas et al 2005) or generalised anxiety disorder (Carlson et al 2004). Therefore, BSI could be used in various populations to determine components such as somatisation, depression, anxiety, phobic anxiety paranoid ideation, interpersonal sensitivity, obsessive-compulsive, anger-hostility, psychoticism. Theoretically, BSI would be quite useful for use among PTB mothers given its clinical relevance (Carlson et al 2004).

The Edinburgh Postnatal Depression Scale (EPDS) is a 10 item questionnaire identifying with depressive and anxiety symptoms elicited during the postnatal and antenatal period. However, EPDS is not a diagnostic tool. It is useful as a screening tool and is beneficial for follow-up care where a psychiatric diagnosis is eminent. It is reported EPDS should be administered at least twice during the antenatal and postnatal (6-12 weeks post-delivery) period. Therefore, some studies have demonstrated a clear scientific rationale for using EPDS at specific timepoints. A disadvantage, however, is that EPDS remains non-specific to BAME populations. There could be cultural adaptations that may be required to make the questionnaire more relevant to BAME groups.

Depression Anxiety Stress Scale (DASS)- 21 is a briefer version of the DASS-42 and is a self-report short questionnaire designed to measure states of depression, tension/stress and anxiety. The stress construct scale in particular is more empirical in nature to identify depression and anxiety by way of aggregating items associated impatience, irritability, restlessness and challenges with relaxing. A key advantage of DASS-21 is that it is multilingual availability and is a preferred method for administering among BAME populations. The psychometric properties could determine MH symptomatologies within clinical and non-clinical populations that display physiological symptoms as well (Silva et al 2016).

Studies have found that the DASS-21 distinguishes well between features of depression, physical arousal, and psychological tension and agitation. In addition, the internal consistency and concurrent validity of the DASS and DASS–21 were in the acceptable to excellent ranges. Cronbach's alphas for the DASS Depression, Anxiety, and Stress subscales were .97, .92, and .95, respectively. Cronbach's alphas for the DASS–21 subscales were .94 for Depression, .87 for Anxiety, and .91 for Stress. Antony, Bieling, Cox, Enns, & Swinson,1998).

The Beck Depression Inventory (BDI) is a self-reported measure with 21 question and is a commonly used inventory to measure severe depression. BDI measures depression beyond the psychodynamic perspective by analysing the patient’s own thoughts and the intensity of the depression syndrome. Therefore, BDI could be used in both clinical and non-clinical populations. Clinical observations using BDI have a rating scale of 0-3.

There is minimal evidence to suggest the administering of these MH questionnaires were of relevance to PTB mother within the BAME population due to its non-specificity, lack of cultural adaptation and evidence demonstrating its validity. As a result, a key recommendation would be to develop better assessment tools for future BAME populations of patients to better determine symptomatologies and deter a clinical diagnosis where appropriate with a view to treat.

PHQ9 is a short but psychometrically valid assessment of depressive symptomatology.

**References**

Lewinsohn, P.M., Seeley, J.R., Roberts, R.E., & Allen, N.B. (1997). Center for Epidemiological Studies-Depression Scale (CES-D) as a screening instrument for depression among community-residing older adults. Psychology and Aging, 12, 277- 287

Radloff, L. S. (1977). The CES-D scale: A self-report depression scale for research in the general population. Applied Psychological Measurements, 1, 385-401

Pinquart, M., & Sorensen, S. (2003). Differences between caregivers and non-caregivers in psychological health and physical health: A meta-analysis. Psychology and Aging, 18, 250–267

Pinquart, M., & Sörensen, S. (2006). Helping caregivers of persons with dementia: Which interventions work and how large are their effects? International Psychogeriatrics, 18, 577-595

Carlson LE, Angen M, Cullum J, Goodey E, Koopmans J, Lamont L, et al. High levels of untreated distress and fatigue in cancer patients. Br J Cancer. 2004;90:2297–304

Coultas D, Frederick J, Barnett B, Singh G, Wludyka P. A randomized trial of two types of nurse-assisted home care for patients with COPD. Chest. 2005;128:2017–24

Franke, G.H., Jaeger, S., Glaesmer, H. *et al.* Psychometric analysis of the brief symptom inventory 18 (BSI-18) in a representative German sample. *BMC Med Res Methodol* **17,**14 (2017)

Silva, Hítalo Andrade da et al. “Short version of the Depression Anxiety Stress Scale-21: is it valid for Brazilian adolescents?.” *Einstein (Sao Paulo, Brazil)* vol. 14,4 (2016): 486-493. doi:10.1590/S1679-45082016AO3732

Lovibond, S.H. & Lovibond, P.F. (1995). Manual for the Depression Anxiety Stress Scales. (2nd. Ed.) Sydney: Psychology Foundation.

[Lovibond, P. F., & Lovibond, S. H. (1995).](https://dionysus.psych.wisc.edu:5001/sharing/q0DfP8UG4) The structure of negative emotional states: Comparison of the Depression Anxiety Stress Scales (DASS) with the Beck Depression and Anxiety Inventories. Behaviour research and therapy, 33(3), 335-343

James N. Butcher, ... G. Cynthia Fekken, in [Comprehensive Clinical Psychology](https://www.sciencedirect.com/referencework/9780080427072/comprehensive-clinical-psychology), 1998

Antony, M. M., Bieling, P. J., Cox, B. J., Enns, M. W., & Swinson, R. P. (1998). Psychometric properties of the 42-item and 21-item versions of the Depression Anxiety Stress Scales in clinical groups and a community sample. *Psychological Assessment, 10*(2), 176-181.

Cho MJ, Kim KH. Use of the Center for Epidemiologic Studies Depression (CES-D) Scale in Korea. J Nerv Ment Dis. 1998 May;186(5):304-10. doi: 10.1097/00005053-199805000-00007. PMID: 9612448.

Demirchyan A, Petrosyan V, Thompson ME. [Psychometric value of the Center for Epidemiologic Studies Depression (CES-D) scale for screening of depressive symptoms in Armenian population.](https://pubmed.ncbi.nlm.nih.gov/21601288/) J Affect Disord. 2011 Oct;133(3):489-98. doi: 10.1016/j.jad.2011.04.042. Epub 2011 May 23.PMID: 21601288

Schantz K, Reighard C, Aikens JE, Aruquipa A, Pinto B, Valverde H, Piette JD. Screening for depression in Andean Latin America: Factor structure and reliability of the CES-D short form and the PHQ-8 among Bolivian public hospital patients. Int J Psychiatry Med. 2017 Jul-Sep;52(4-6):315-327. doi: 10.1177/0091217417738934. Epub 2017 Nov 6. PMID: 29108457.

Baron EC, Davies T, Lund C. Validation of the 10-item Centre for Epidemiological Studies Depression Scale (CES-D-10) in Zulu, Xhosa and Afrikaans populations in South Africa. BMC Psychiatry. 2017 Jan 9;17(1):6. doi: 10.1186/s12888-016-1178-x. PMID: 28068955; PMCID: PMC5223549.

Edge, D., Baker, D., Rogers, A., Perinatal depression among black Caribbean women

Health and Social Care in the community, 19 August 2004 <https://doi.org/10.1111/j.1365-2524.2004.00513.x>

Uwakwe, R (2003). Affective (depressive) morbidity in puerperal Nigerian women: validation of the Edinburgh postnatal depression scale. Acta Psychiatrica Scandinavica, 27 March 2003; <https://doi.org/10.1034/j.1600-0447.2003.02477.x>
